# Multisensory gamma stimulation promotes glymphatic clearance of amyloid

**DOI:** 10.1038/s41586-024-07132-6

**Published:** 2024-02-28

**Authors:** Mitchell H. Murdock, Cheng-Yi Yang, Na Sun, Ping-Chieh Pao, Cristina Blanco-Duque, Martin C. Kahn, TaeHyun Kim, Nicolas S. Lavoie, Matheus B. Victor, Md Rezaul Islam, Fabiola Galiana, Noelle Leary, Sidney Wang, Adele Bubnys, Emily Ma, Leyla A. Akay, Madison Sneve, Yong Qian, Cuixin Lai, Michelle M. McCarthy, Nancy Kopell, Manolis Kellis, Kiryl D. Piatkevich, Edward S. Boyden, Li-Huei Tsai

**Affiliations:** 1 Department of Brain and Cognitive Sciences and the Picower Institute for Learning and Memory, Massachusetts Institute of Technology, Cambridge, MA USA; 2 MIT Computer Science and Artificial Intelligence Laboratory, Cambridge, MA USA; 3 Broad Institute of MIT and Harvard, Cambridge, MA USA; 4 Departments of Biological Engineering and Brain and Cognitive Sciences, McGovern Institute, Cambridge, MA USA; 5 Koch Institute, Massachusetts Institute of Technology, Cambridge, MA USA; 6 Howard Hughes Medical Institute, Massachusetts Institute of Technology, Cambridge, MA USA; 7 School of Life Sciences, Westlake University, Westlake Laboratory of Life Sciences and Biomedicine, and Westlake Institute for Advanced Study, Hangzhou, China; 8 Department of Mathematics and Statistics, Boston University, Boston, MA USA

**Keywords:** Cellular neuroscience, Neuro-vascular interactions, Alzheimer's disease

## Abstract

The glymphatic movement of fluid through the brain removes metabolic waste^1–4^. Noninvasive 40 Hz stimulation promotes 40 Hz neural activity in multiple brain regions and attenuates pathology in mouse models of Alzheimer’s disease^5–8^. Here we show that multisensory gamma stimulation promotes the influx of cerebrospinal fluid and the efflux of interstitial fluid in the cortex of the 5XFAD mouse model of Alzheimer’s disease. Influx of cerebrospinal fluid was associated with increased aquaporin-4 polarization along astrocytic endfeet and dilated meningeal lymphatic vessels. Inhibiting glymphatic clearance abolished the removal of amyloid by multisensory 40 Hz stimulation. Using chemogenetic manipulation and a genetically encoded sensor for neuropeptide signalling, we found that vasoactive intestinal peptide interneurons facilitate glymphatic clearance by regulating arterial pulsatility. Our findings establish novel mechanisms that recruit the glymphatic system to remove brain amyloid.

## Abstract (web summary)

Audio and visual stimulation at 40 Hz promote cerebrospinal and interstitial fluid flux in mouse brain and result in amyloid clearance via the glymphatic system in a mouse model of Alzheimer’s disease.

## Main

The glymphatic system is thought to have a critical role in clearing metabolic waste from the brain^1^. According to the glymphatic model, arterial pulsation drives cerebrospinal fluid (CSF) along an interconnected network of perivascular spaces surrounding blood vessels in the brain^2,9^, and the water channel aquaporin-4 (AQP4) localized on astrocytic endfeet facilitates exchange between CSF and interstitial fluid (ISF)^2,10^. Glymphatic transport has been shown to clear parenchymal metabolites, including pathogenic proteins such as amyloid^3,11^. Ageing and Alzheimer’s disease impair the fitness of glymphatic clearance by attenuating arterial pulsation^12^, reducing CSF–ISF exchange^11,13^, and diminishing lymphatic drainage, leading to the pathogenic accumulation of amyloid and tau. Therapeutic interventions to promote glymphatic clearance have been hypothesized to alleviate Alzheimer’s disease pathology^14^.

In previous studies, we found that 1 h of optogenetic-driven 40 Hz stimulation attenuated pathogenic amyloid burden in a mouse model of Alzheimer’s disease compared with non-gamma control stimulations^5^. To extend the clinical translatability of this intervention, we explored noninvasive approaches to induce 40 Hz neural activity. In humans and mice, sensory stimuli at specific frequencies can be used to promote neural activity corresponding to the sensory stimulation^15^. Noninvasive 40 Hz stimulation promotes 40 Hz neural activity in multiple brain regions, including the prefrontal cortex^6,7,16^, and we and others found that noninvasive 40 Hz sensory stimulation attenuates amyloid burden in Alzheimer’s disease model mice^8^. In particular, multisensory 40 Hz stimulation (that is, combined light and sound stimulation) attenuates amyloid burden throughout the cortex, including the prefrontal cortex^6^.

Brain metabolites are removed via glial-mediated phagocytosis and vascular-mediated clearance^17^. It has been unclear whether 40 Hz stimulation attenuates amyloid burden through glymphatic routes. Previous reports suggest that CSF influx to the brain is increased during anaesthesia and sleeping^1,18^, indicating that distinct brain rhythms influence glymphatic transport. Arteriolar vasomotion, which drives CSF influx^2,9^ and paravascular clearance^19^, is coupled to gamma oscillations^20^. We hypothesized that noninvasive gamma stimulation may clear amyloid in part via glymphatic transport.

## Gamma stimulation promotes glymphatic clearance

**Fig. 1:**
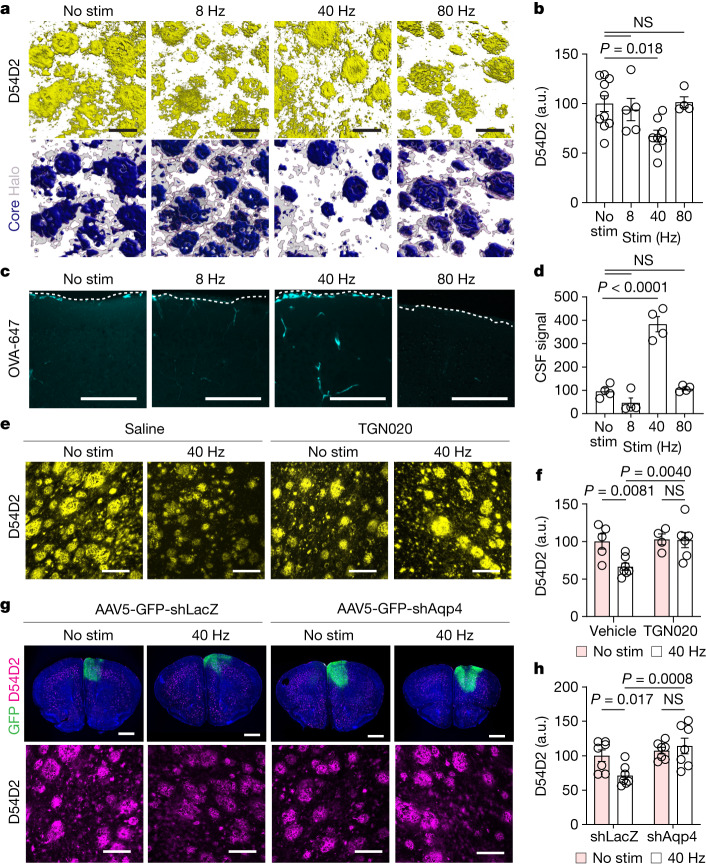
Multisensory 40 Hz stimulation promotes AQP4-dependent clearance of amyloid. **a**, Top row, example confocal *z*-stack reconstructions of D54D2 (monoclonal β-amyloid antibody) signal from frontal cortex of 6-month-old 5XFAD mice with stimulations at indicated frequency or no stimulation (No stim). Bottom row, amyloid signals based on dense-core and non-core regions. Scale bars, 20 μm. **b**, Quantification of D54D2 signal intensity in experiments represented in **a** (*n* = 10 (no stimulation), 5 (8 Hz), 8 (40 Hz) and 4 (80Hz) 6-month-old 5XFAD mice; *P* values by one-way analysis of variance (ANOVA) and Dunnett’s multiple comparison test). a.u., arbitrary units; NS, not significant. **c**, Maximum intensity projections of confocal *z*-stacks for cisterna magna-infused CSF tracer (OVA-647) imaged in frontal cortex. The dotted line shows cortical surface. Scale bars, 100 μm. **d**, Quantification of cisterna magna-infused OVA-647 from **c** (*n* = 4 6-month-old 5XFAD mice per condition; *P* values by one-way ANOVA and Dunnett’s multiple comparison test). **e**, Example confocal *z*-stack projections of D54D2 in frontal cortex of 6-month-old 5XFAD mice. Scale bars, 100 μm. **f**, Quantification of amyloid signal intensity in frontal cortex (*n* = 5 (no stimulation), 7 (40 Hz), 4 (no stimulation + TGN020) and 6 (40 Hz + TGN020) 6-month-old 5XFAD mice; *P* values by two-way ANOVA and Fisher’s least significant difference (LSD) test). **g**, Top row, example coronal section showing signal from Hoechst (blue), adeno-associated virus (AAV) expressing short hairpin RNA (shRNA) targeting *Aqp4* (AAV-eGFP-shAqp4) or *lacZ* (AAV-eGFP-shLacZ) and D54D2. Bottom row, example confocal *z*-stack maximum intensity projection of D54D2 signal. Scale bars: 1,000 μm (top), 100 μm (bottom). **h**, Quantification of D54D2 signal intensity from experiments represented in **g** (*n* = 6 (no stimulation), 7 (no stimulation + shLacZ), 7 (40 Hz + shLacZ) and 7 (40 Hz + shAqp4); *P* values by two-way ANOVA and Fisher’s LSD test; data are mean ± s.e.m.). Source Data

**Extended Data Fig. 1:**
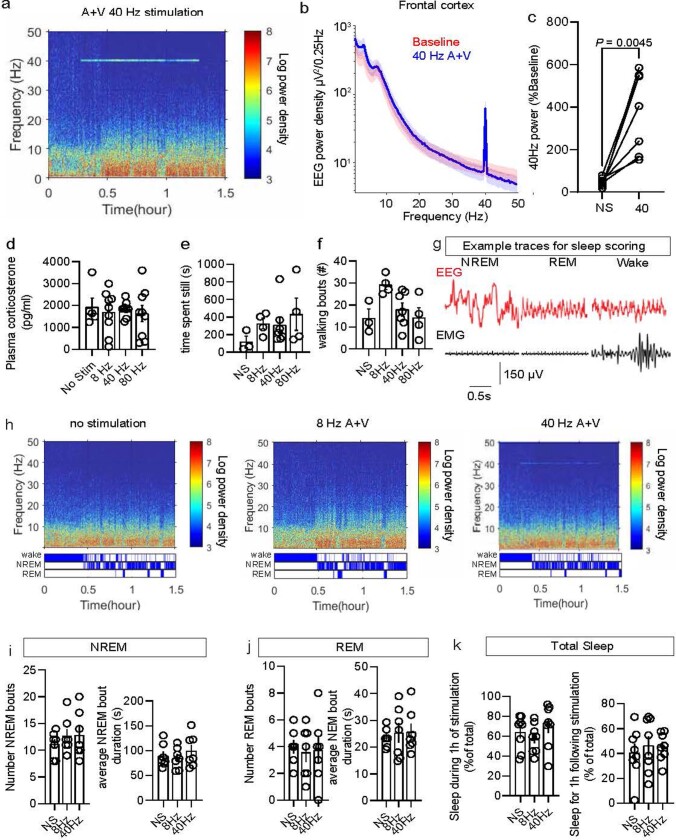
Arousal state following multisensory stimulations in 6-month-old 5XFAD mice. a. Example spectrogram from frontal EEG signal recorded in one 6-month-old 5XFAD mouse during 40 Hz stimulation (presented between 0.3 h and 1.3 h). b. Mean EEG power density measured in frontal cortex (*n* = 7 6-month-old 5XFAD mice; data is presented as the mean across all mice; shaded area represents the standard error of the mean). c. Average power at 40 Hz measured in frontal cortex of 6-month-old 5XFAD mice during one hour of audio-visual 40 Hz stimulation compared to baseline period (no stimulation). Values are expressed as % of mean power density during baseline (no stimulation) (*n* = 7 6-month-old 5XFAD mice; data is presented as the mean ± s.e.m.; P value was calculated by paired two-tailed t-test). d. Plasma corticosterone measured by ELISA following 1 h of multisensory stimulation (either 8 Hz, 40 Hz, or 80 Hz multisensory stimulation or no stimulation) in 6-month-old 5XFAD mice (*n* = 5 mice for no stimulation; 9 mice for 8 Hz; 9 mice for 40 Hz; 9 mice for 80 Hz; data is presented as the mean ± s.e.m.; statistical analysis used one-way ANOVA followed by Tukey’s multiple comparisons test). e. Time spent still (seconds) during 1 h of multisensory stimulation (either no stimulation, 8 Hz, 40 Hz, or 80 Hz multisensory stimulation) in 6-month-old 5XFAD mice (n = 3 mice for no stimulation (NS); 4 mice for 8 Hz; 7 mice for 40 Hz; 4 mice for 80 Hz; data is presented as the mean ± s.e.m.; one-way ANOVA followed by Tukey’s multiple comparisons test was used for statistical analysis). f. Number of walking bouts during 1 h of multisensory stimulation (either no stimulation, 8 Hz, 40 Hz, or 80 Hz multisensory stimulation) in 6-month-old 5XFAD mice (n = 3 mice for no stimulation (NS); 4 mice for 8 Hz; 7 mice for 40 Hz; 4 mice for 80 Hz; data is presented as the mean ± s.e.m.; one-way ANOVA followed by Tukey’s multiple comparisons test was used for statistical analysis). g. Representative EEG traces (recorded from the frontal cortex) and the EMG (recorded from the neck muscle) during NREM, REM and wake in one mouse using established methods^54^. h. Representative spectrograms (top) and hypnogram (bottom) for each stimulation condition in one mouse. The top bar of the hypnogram represents the time spent wake, middle bar represents NREM state, and bottom bar represents REM state. i. Quantification of NREM bouts (left) and average NREM bout duration (right) across stimulation conditions (no stimulation; 8 Hz; or 40 Hz) (*n* = 7 6-month-old 5XFAD mice; repeated measures paired one-way ANOVA followed by Tukey’s multiple comparisons test was used for statistical analysis; data is presented as the mean ± s.e.m.). j. Quantification of REM bouts (left) and average REM bout duration (right) across stimulation conditions (no stimulation; 8 Hz; or 40 Hz) (*n* = 7 6-month-old 5XFAD mice; repeated measures paired one-way ANOVA followed by Tukey’s multiple comparisons test was used for statistical analysis; data is presented as the mean ± s.e.m.). k. Quantification of total sleep during (left) and 1 h after (right) stimulation (No stimulation (NS), 8 Hz audio-visual, 40 Hz audio-visual stimulation) as a percent of total time (*n* = 8 6-month-old 5XFAD mice; repeated measures paired one-way ANOVA followed by Tukey’s multiple comparisons test was used for statistical analysis; data is presented as the mean ± s.e.m.). Source Data

We found that 40 Hz multisensory audio-visual stimulation increased 40 Hz local field potential power in frontal cortex areas of 6-month-old 5XFAD mice (Extended Data Fig. 1a–c), as expected on the basis of prior reports in mice^6^ and humans^8,16^. In separate cohorts of 6-month-old 5XFAD mice, 40 Hz stimulation attenuated amyloid burden compared with no stimulation, 8 Hz stimulation and 80 Hz stimulation controls (Fig. 1a,b). Reductions in amyloid by 40 Hz multisensory stimulation were not associated with alterations in plasma corticosterone (Extended Data Fig. 1d), time spent moving (Extended Data Fig. 1e–f) or sleep architecture (Extended Data Fig. 1g,h), including neither rapid eye movement (REM) nor non-REM (NREM) sleep states (Extended Data Fig. 1i–k).

**Extended Data Fig. 2:**
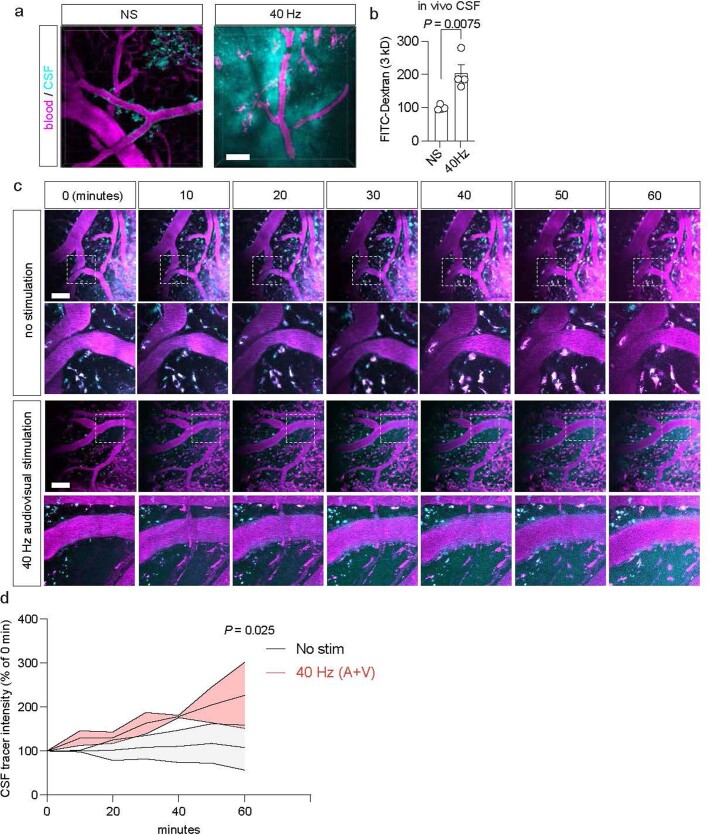
Monitoring CSF dynamics following multisensory 40 Hz stimulation via two-photon microscopy in awake 6-month-old 5XFAD mice. a. Example 2 P *z*-stacks of vascular blood (magenta, Texas Red-Dextran-70kD, injected retro-orbitally) and CSF tracer (cyan, FITC-Dextran-3kD, infused through a cannula installed into the cisterna magna) following 1 h of multisensory gamma stimulation or no stimulation control, imaged via a cranial window in frontal cortex. Scale bar, 50 μm. b. Quantification of CSF tracer (FITC-Dextran-3kD) infused via intracisternal cannulation into 6-month-old 5XFAD mouse cortex via 2 P microscopy. The intensity of tracer signal was quantified using ImageJ (*n* = 4 mice per condition; each data point represents the mean of at least two z-stacks per mouse; data is presented as the mean ± s.e.m.; *P* value represents unpaired two-sided t-test). c. Example 2 P z-stacks imaged every 10 min after no stimulation or 40 Hz stimulation. Images are representative of two separate experiments. Scale bar, 100 um. d. Quantification of CSF tracer dynamics (n = 2 6-month-old 5XFAD mice per group; *P* value by two-way ANOVA followed by Fisher’s LSD test). Source Data

**Extended Data Fig. 3:**
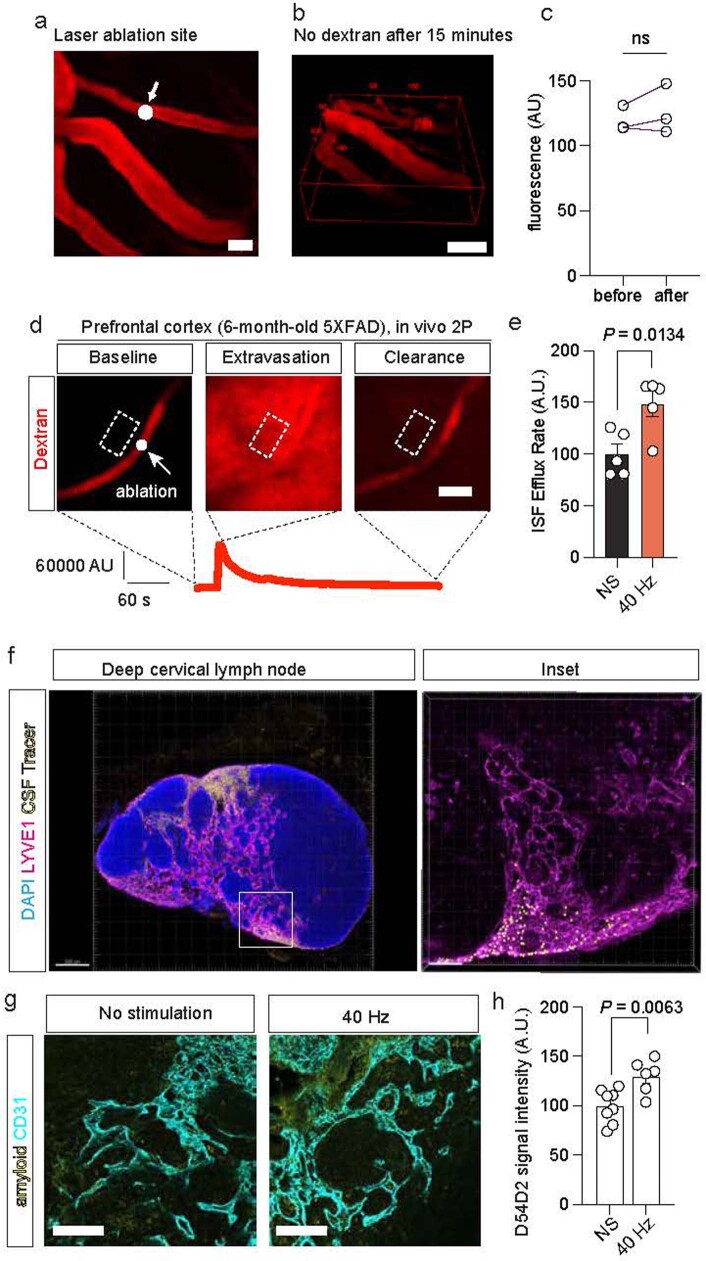
Clearance assays following gamma stimulation. a. The white dot marks the representative position of a 2P-laser ablation applied to a vascular segment, to induce brief extravasation of intravenously loaded dextran (Texas Red-Dextran-70kD). Scale bar, 20 μm. b. Representative volumetric scan of Texas Red-Dextran-70kD in mouse PFC 15 min after application of the laser ablation, suggesting extravasated tracer is cleared within 15 min. c. Quantification of extravascular fluorescence before and after ISF ablation assay (*n* = 3 regions of interest from 1 6-month-old 5XFAD mouse; two-tailed unpaired t-test was used for statistical analysis). Scale bar, 50 um. d. Example images of 2 P focal ablation assay showing clearance of extravasated material due to interstitial fluid flux in 6-month-old 5XFAD mice. The white dot (left panel) marks the representative position of a 2P-laser ablation applied to a vascular segment, inducing extravasation of intravenously loaded dextran (Texas Red-Dextran-70kD; middle panel) that is then quickly cleared from the brain parenchyma (right panel; scale bar, 10 μm). The red graph under the panel shows the Texas Red fluorescence intensity in the region surrounded by the dotted box over time. e. Quantification of interstitial fluid efflux in 6-month-old 5XFAD mice following 1 h of multisensory gamma stimulation or non-stimulated control (*n* = 5 6-month-old 5XFAD mice per stimulation condition; each mouse was imaged in 3 vessel segments; data is presented as the mean ± s.e.m.; *P* value by unpaired two-tailed student’s t-test). f. Example confocal image of lymph node following infusion of FluoSphere beads to the cisterna magna. Inset reveals beads in LVYE+ area. Scale bar, 200 μm. This experiment was performed once. g. Example confocal z-stack projections of endothelial cells (CD31, cyan) and amyloid (D54D2, yellow) in deep cervical lymph nodes from 6-month-old 5XFAD mice receiving 1 h of multisensory gamma stimulation or no stimulation. Scale bar. h. Quantification of amyloid in deep cervical lymph nodes in 6-month-old 5XFAD mice after no stimulation (NS) or 1 h of 40 Hz stimulation (*n* = 8 mice for no stimulation and 7 mice for 40 Hz stimulation; each data point represents the mean signal intensity from confocal *z*-stacks acquired from at least three lymph nodes from each mouse; data is presented as the mean ± s.e.m.; *P* value calculated by unpaired two-tailed student’s t-test). Source Data

To test whether glymphatic clearance is associated with multisensory 40 Hz-mediated amyloid clearance, we began by monitoring CSF dynamics. We infused a fluorescent tracer (ovalbumin–Alexa Fluor 647 (OVA-647), with a molecular mass of 45 kDa) to the cisterna magna of 6-month-old 5XFAD mice, presented multisensory stimulation, and then evaluated tracer movement into the cortex using coronal sections and confocal microscopy. Given evidence highlighting changes in CSF dynamics related to circadian rhythms^21^, arousal state^1,18^, tracer infusion method^22^ and histological preparations^23^, we maintained identical preparations between treatment groups. We observed increased CSF tracer accumulation in the cortex following 1 h of multisensory 40 Hz multisensory stimulation compared with no stimulation, 8 Hz multisensory stimulation and 80 Hz multisensory stimulation controls (Fig. 1c,d). As an orthogonal approach, we obtained volumetric scans of the cortex using two-photon microscopy via cranial windows installed over the prefrontal cortex at least 3 weeks prior, and found that multisensory 40 Hz stimulation increased CSF tracer influx (FITC–dextran, 3 kDa) into the cortex (Extended Data Fig. 2a–d and Supplementary Video 1). To estimate possible relative differences in ISF efflux between gamma stimulation and non-stimulation control, we exposed 6-month-old 5XFAD mice to 1 h of multisensory gamma stimulation then measured ISF efflux rates via clearance of extravasated fluorescent dextran in the cortex^19^ (Extended Data Fig. 3a–d) and found that the ISF efflux rate increased in 6-month-old 5XFAD mice that received multisensory 40 Hz stimulation (Extended Data Fig. 3e). These results suggest that multisensory 40 Hz stimulation increases the clearance rate of ISF. Soluble amyloid is thought to be cleared from the brain and collected in part via meningeal lymphatic vessels^4,11^, which drain to deep cervical lymph nodes^24^. We found that cervical lymph nodes drain CSF macromolecules (Extended Data Fig. 3f), and multisensory 40 Hz stimulation increased amyloid accumulation in cervical lymph nodes (Extended Data Fig. 3g,h). These findings indicate that amyloid is cleared from the brain following multisensory 40 Hz stimulation in part via glymphatic routes.

**Extended Data Fig. 4:**
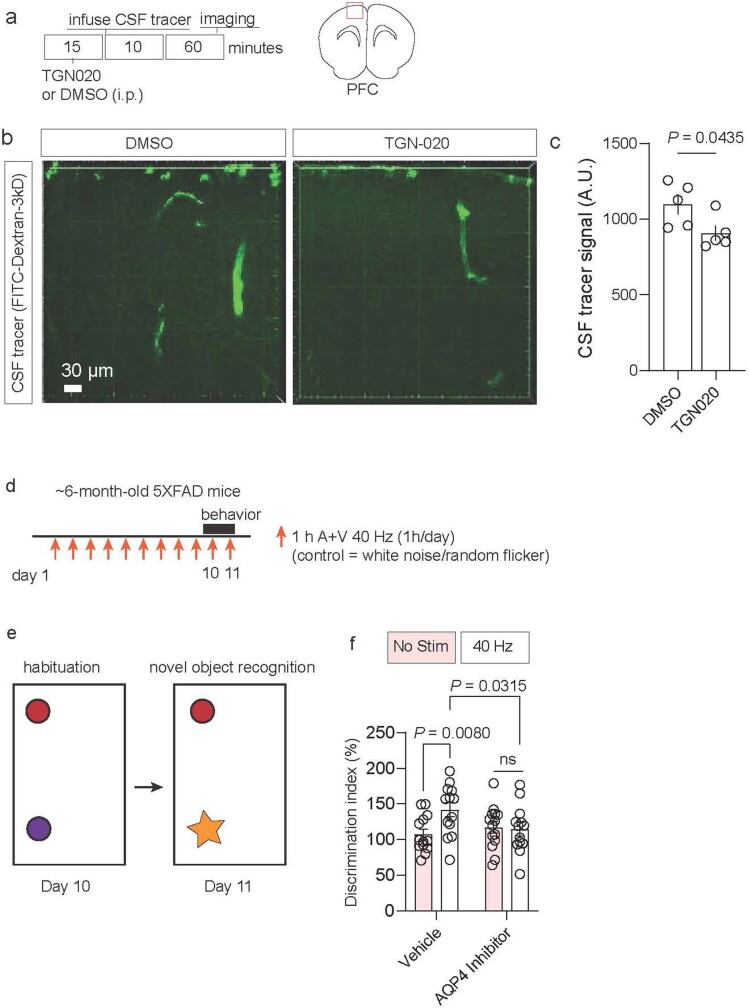
Effects of TGN020 on CSF tracer dynamics and behavior. a. Experimental schematic. C57BL6/J mice received intraperitoneal injection of TGN020 or DMSO control. CSF tracer was infused via intracisternal magna and tracer was allowed to circulate for 60 min. b. Example images of CSF tracer (FITC-3kD-dextran) infused to cisterna magna and quantified in frontal cortex. Scale bar, 30 μm. c. Quantification of CSF tracer signal in frontal cortex (n = 5 C57BL6/J mice per group; data presented as mean +/− SEM; *P* value was calculated by unpaired two-tailed student’s t-test). d. Experimental schematic. 6-month-old 5XFAD mice were subjected to daily (1 h/day) of audio + visual 40 Hz stimulation (control = white noise and random flicker at the same lux and sound intensity). Mice received i.p. injections of vehicle or TGN020 prior to daily stimulation. e. Novel object recognition testing was performed on days 10 and 11. f. Quantification of discrimination index following novel object recognition test quantified as percent of time spent exploring novel object (n = 13 6-month-old 5XFAD mice for Vehicle + No Stim, 14 animals for Vehicle + 40 Hz; 13 mice for TGN020 + No Stim; 13 mice for TGN020 + 40 Hz. P values represent two-way ANOVA followed by Fisher’s LSD multiple comparison’s test; data is presented as the mean ± s.e.m.). Source Data

**Extended Data Fig. 5:**
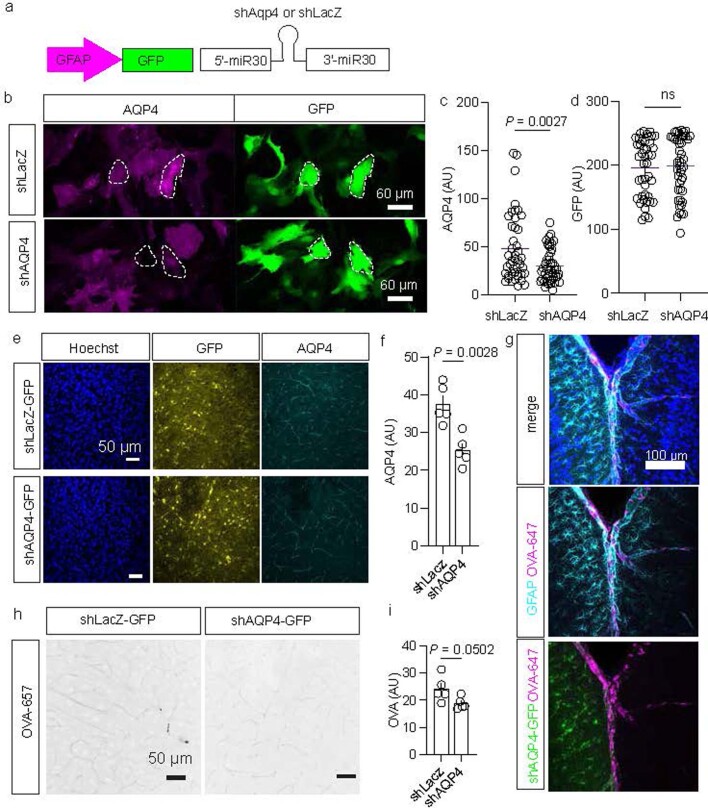
shAQP4 strategy to attenuate AQP4 function. a. miR-30 based shRNA strategy to reduce AQP4. Constructs target the coding region of AQP4 or, as a control, LacZ. Constructs targeting three different regions of AQP4 were packaged into individual AAVs and then pooled for injection. b. Example confocal z-stack maximum intensity projections of mouse primary astrocyte cultures showing AQP4 signal (magenta) and GFP (green) following AAV application delivering shAQP4 or shLacZ. c. Quantification of AQP4 signal intensity (n = 43 cells for shLacZ, n = 46 cells for shAQP4; data is presented as the mean ± s.e.m; *P* value calculated by unpaired two-tailed student’s t-test). d. Quantification of GFP signal intensity (n = 43 cells for shLacZ, n = 46 cells for shAQP4; data is presented as the mean ± s.e.m; unpaired two-tailed student’s t-test was used for statistical analysis). e. Example confocal z-stack maximum intensity projections of mouse cortex following 4 weeks of viral injection (AAV-GFAP-shLacZ or AV-GFAP-shAQP4-GFP). Immunostaining shows Hoechst (blue), AQP4 signal (cyan) and GFP (yellow). Scale bar, 50 um. f. Quantification of AQP4 signal intensity from z-stacks. (n = 5 3-month-old C57BL/6 J mice for shLacZ and 5 mice for shAQP4; data is presented as the mean ± s.e.m; *P* value calculated by unpaired two-tailed student’s t-test). g. Example confocal z-stack maximum intensity projections of 3-month-old C57BL/6 J mouse cortex following 4 weeks of viral injection (AAV-GFAP-shLacZ or AV-GFAP-shAQP4-GFP) followed by OVA-647 injection to the cisterna magna. Immunostaining shows Hoechst (blue), GFP signal (green) and OVA-647 CSF tracer (magenta). This experiment was performed twice. Scale bar, 50 um. h. Example confocal z-stack maximum intensity projections of mouse cortex of OVA-47 (black). i. Quantification of OVA CSF tracer in mouse cortex after shLacZ or shAQP4 (n = 5 mice for shLacZ and 5 mice for shAQP4; data is presented as the mean ± s.e.m; *P* value calculated by unpaired two-tailed student’s t-test). Source Data

To causally test the hypothesis that multisensory 40 Hz stimulation recruits glymphatic systems to promote amyloid clearance, we manipulated the astrocytic water channel AQP4, owing to its reported role in glymphatic transport^10^. Consistent with prior reports^13,25^, we found that impairing AQP4 function using the small molecule TGN020 reduced CSF tracer influx into the brain (Extended Data Fig. 4a–c). TGN020 attenuated multisensory 40 Hz-mediated amyloid clearance (Fig. 1e,f). Notably, TGN020 pretreatment also appeared to attenuate the effect of chronic daily multisensory 40 Hz effects on cognitive performance tested in novel object recognition (Extended Data Fig. 4d–f), although we cannot rule out a possible behavioural effect of chronic TGN020 treatment^6^. As an additional method to modulate AQP4-dependent glymphatic function, we genetically attenuated *Aqp4* in astrocytes^26^, which we confirmed reduced AQP4 in primary astrocytes (Extended Data Fig. 5a–d) and mouse brain (Extended Data Fig. 5e–i). Genetic reduction of *Aqp4* attenuated multisensory 40 Hz stimulation-mediated amyloid clearance in 5XFAD mice (Fig. 1g,h). We note that the prolonged effect of AQP4 reduction exacerbated amyloid burden, and that the TGN020 experiment may not have exacerbated amyloid burden owing to the acute nature of the experiment. Collectively, these results highlight a role of AQP4-dependent glymphatic clearance of amyloid by multisensory 40 Hz stimulation.

## Gamma stimulation promotes arterial pulsation

**Fig. 2:**
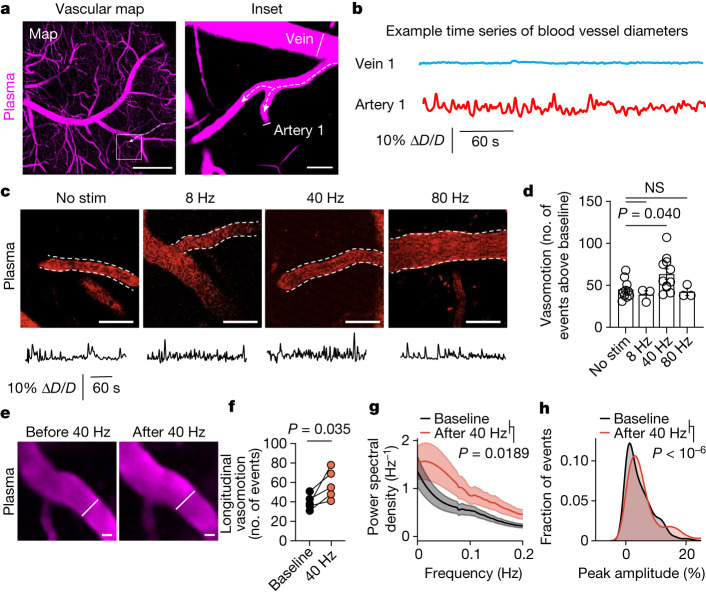
Multisensory 40 Hz stimulation promotes arterial pulsatility. **a**, Example image of blood vessels imaged by two-photon microscopy through a cranial window using Texas Red–dextran 70 kD in 6-month-old 5XFAD mice. Dotted arrows depict blood flow direction, and line segments indicate regions used to quantify blood vessel diameter over time. Scale bars: 500 μm (left) and 20 μm (right). **b**, Example time series of diameter (*D*) of blood vessels. ∆*D*/*D* is change in blood vessel diameter as a fraction of the mean diameter. **c**, Example images and traces of vasomotion after multisensory stimulations in 6-month-old 5XFAD mice. Scale bars, 50 μm. **d**, Number of peaks after 1 h of noninvasive multisensory stimulation in 6-month-old 5XFAD mice (*n* = 10 (no stimulation), 3 (8 Hz), 11 (40 Hz) and 3 (80 Hz) vascular segments; *P* values by one-way ANOVA followed by Dunnett’s multiple comparisons test; data are mean ± s.e.m.). **e**, Repeated imaging of the arterial segments before and after 1 h of noninvasive multisensory gamma stimulation. Example images and representative diameter traces are shown. Scale bars, 20 μm. **f**, Number of peaks within time series traces from vasomotion patterns for a subset of mice from **d** imaged longitudinally (*n* = 5 mice imaged before and after 40 Hz stimulation; *P* values by paired *t*-test). **g**, Fast Fourier transform analysis of arterial vasomotion (*n* = 5 mice imaged both before and after gamma stimulation; *P* values by paired *t*-test; shaded areas represent s.e.m.). **h**, Distribution of vasomotive events based on amplitude peak over baseline (*n* = 5 mice imaged both before and after gamma stimulation; *P* < 10^−6^ by two-tailed Mann–Whitney test). Source Data

**Extended Data Fig. 6:**
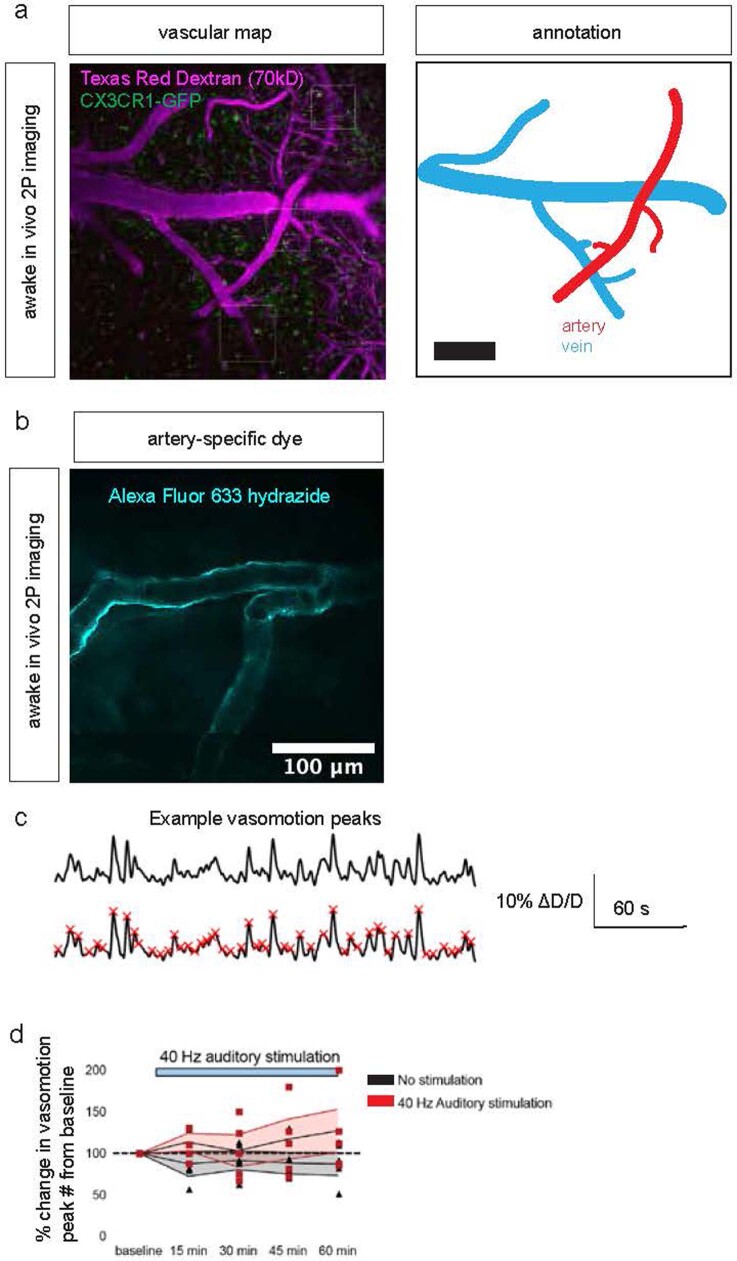
Validation of arterial imaging, including arterial segment identification and motion correction. a. Example two-photon image of blood (magenta) and microglia (green) to define vascular segments. This experiment annotating vascular segments was used for all 2 P imaging of vasculature. b. Example image of Alexa-633, an artery marker, to define arterial segments. The experiment was repeated twice. Scale bar, 100 μm. c. Example quantification of vasomotion peaks. A Savitzky-Golay filter was applied to the vasomotion trace, and peaks were identified using find_peaks (scipy.signal). d. Longitudinal imaging of 40 Hz auditory stimulation and simultaneous 2 P vasomotion analysis in frontal cortex of 6-month-old 5XFAD mice normalized to baseline at the first imaging session prior to the onset of multisensory 40 Hz stimulation (*n* = 5 6-month-old 5XFAD mice; data is presented as the mean ± s.e.m). Source Data

Arterial vasomotion regulates CSF movement^2,9^ and is entrained by the envelope of gamma rhythms^20^. Thus, we explored whether changes in CSF influx by multisensory 40 Hz stimulation may be owing to arterial pulsatility. We used Texas Red–dextran (70 kD) to label blood vessels in 6-month-old 5XFAD mice with cranial windows over the prefrontal cortex. After habituating mice to head fixation and two-photon imaging, we identified arteries on the basis of blood flow direction, morphology and Alexa 633 hydrazide labelling^27^ (Fig. 2a,b and Extended Data Fig. 6a,b). We imaged changes in arterial diameter in awake mice over 5-min recordings, validating previous reports^19,20^ that indicate oscillatory dynamics in arteries but not in veins (Fig. 2b and Supplementary Video 2), probably reflecting a combination of cell-autonomous oscillator properties and neurovascular coupling dynamics. To evaluate whether gamma stimulation increases arterial pulsatility in awake mice, we administered multisensory stimulation for 1 h, then imaged arterial vasomotion (Fig. 2c). After quantifying arterial vasomotion peaks (Extended Data Fig. 6c), we found that multisensory 40 Hz stimulation increased arterial pulsations compared with non-40 Hz control stimulations (Fig. 2d). Repeated imaging of the same arterial segments (Fig. 2e) revealed that multisensory 40 Hz stimulation increased high-amplitude vasomotor events (Fig. 2f) and power in the band around 0.1 Hz (Fig. 2g), and that gamma stimulation shifted vasomotor events to increased amplitude pulsations (Fig. 2h). We speculate that stimulation itself may increase vasomotion during the stimulation period (Extended Data Fig. 6d), and we attribute the sustained increase in vasomotion following the end of stimulation to the long-lasting effects of sustained peptide signalling on vascular changes.

**Extended Data Fig. 7:**
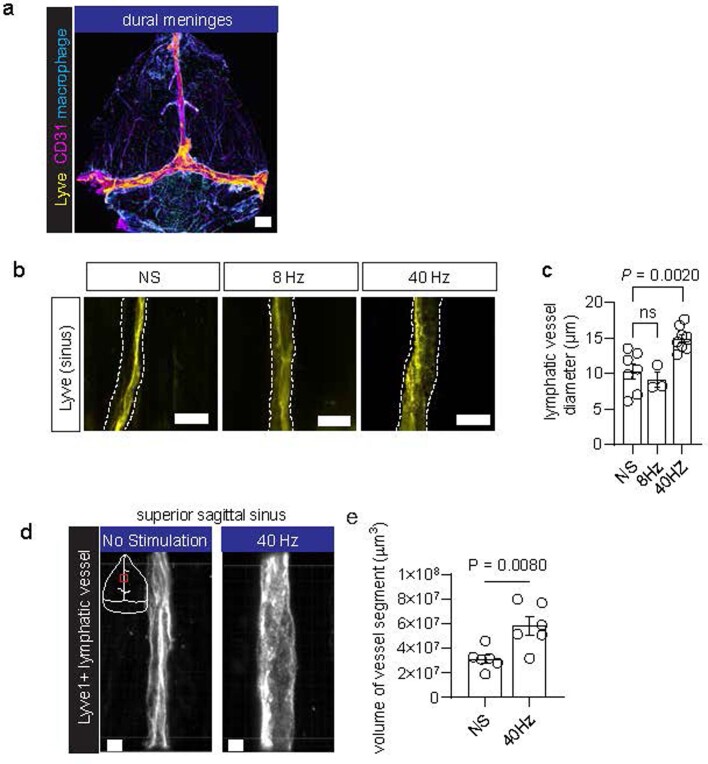
Meningeal lymphatic response to multisensory 40 Hz. a. Example confocal tile z-stack image of a whole mount preparation of the dural meninges showing endothelial cells (CD31, magenta), lymphatic endothelial cells (LYVE1, yellow), and meningeal macrophages (Lyve1 non-vascular cells, cyan). Dural meninges were obtained from the skull caps of 6-month-old 5XFAD mice. Similar dural whole mounts were obtained for all images of the sinus regions. Scale bar, 1000 μm. b. Example confocal images of lymphatic vessels in the dural meninges in 6-month-old 5XFAD mice. c. Quantification of diameter of meningeal lymphatic vessel using confocal microscopy in 6-month-old 5XFAD mice (*n* = 7 mice for no stimulation, 3 mice for 8 Hz stimulation, and 8 mice for 40 Hz stimulation; each data point represents the mean lymphatic vessel diameter from 3 images of the superior sagittal sinus; data is presented as the mean ± s.e.m.; *P* value calculated by one-way ANOVA followed by Dunnett’s multiple comparisons test). Scale bar, 20 μm. d. Example Airyscan confocal images of LYVE1+ lymphatic vessels in the meningeal superior sagittal sinus region (red box in the white schematic of the dural meninges) from 6-month-old 5XFAD mice receiving either 40 Hz stimulation or no stimulation. 3D images of z-stacks from lymphatic vessel segments were generated using Imaris. Scale bar, 5 μm. e. Quantification of lymphatic vessel volume in 6-month-old 5XFAD mice receiving 40 Hz or no stimulation (NS) (n = 6 mice per condition; each data point represents the mean volume from 3 segments of meningeal lymphatic vessel from the superior sagittal sinus for each mouse; data is presented as the mean ± s.e.m.; *P* value calculated by unpaired student’s two-tailed t-test). Source Data

Given that meningeal lymphatic vessels drain CSF^24^ and that amyloid clearance is facilitated by meningeal lymphatic drainage^4,11^, we hypothesized that meningeal lymphatic vessels may participate in glymphatic exchange. Using whole mounts of the dural meninges, we found meningeal lymphatic vessels along the central sinuses, in accord with previous reports^24^ (Extended Data Fig. 7a). We found that multisensory 40 Hz stimulation increased meningeal lymphatic vessel diameter compared with control stimulation in 6-month-old 5XFAD mice (Extended Data Fig. 7b,c). High-resolution volumetric 3D reconstruction analysis revealed that the volume of lymphatic vessels increased following multisensory 40 Hz stimulation (Extended Data Fig. 7d,e and Supplementary Video 3). These results suggest that gamma stimulation increases lymphatic diameter in the dural meninges.

## Gamma stimulation modulates astrocytic endfeet

**Fig. 3:**
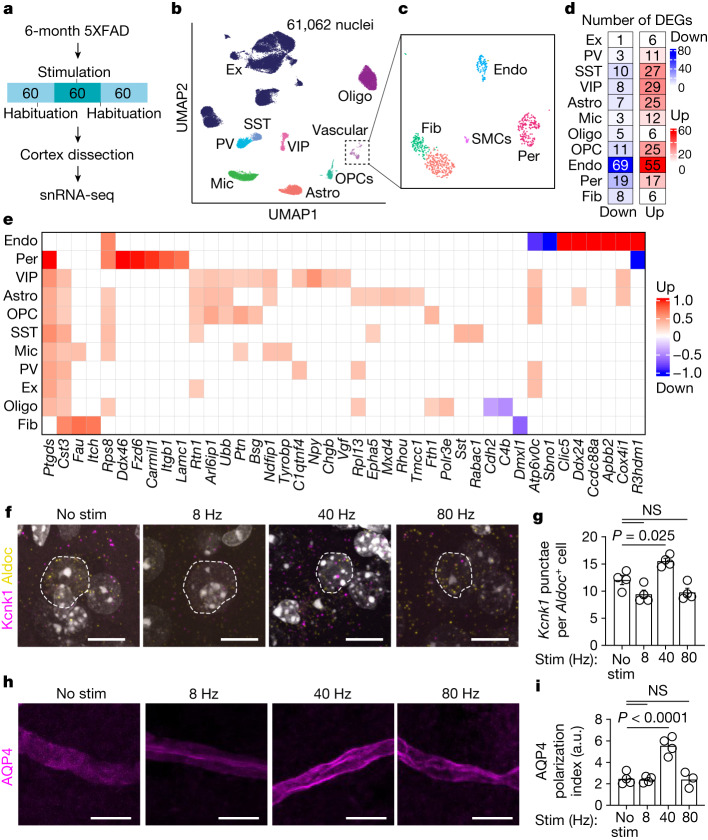
snRNA-seq of mouse cortex following gamma stimulation reveals changes in astrocyte membrane trafficking. **a**, Six-month-old 5XFAD mice were presented with 1 h of 40 Hz multisensory stimulation or no stimulation, allowed to rest for 1 h, and cortex was prepared for snRNA-seq. **b**, Cell type clustering by uniform manifold approximation and projection (UMAP) of 61,062 high-quality nuclei. Cells were classified as excitatory neurons (Ex), parvalbumin interneurons (PV), somatostatin interneurons (SST), vasoactive intestinal peptide (VIP) interneurons, microglia (Mic), astrocytes (Astro), oligodendrocyte precursor cells (OPCs), oligodendrocytes (Oligo) and vascular cells. **c**, Vascular cells were annotated using in silico enrichment as endothelial cells (Endo), smooth muscle cells (SMCs), fibroblasts (Fib) and pericytes (Per). **d**, Differentially expressed genes (DEGs) with 1 h of 40 Hz stimulation versus no stimulation for each cell type. **e**, DEGs per cell type based on fold-change difference with stimulation. **f**, Example confocal *z*-stack projections of RNA in situ hybridization of the DEG *Kcnk1* (magenta) in astrocyte-specific nuclei (*Aldoc*^+^, yellow). Dashed white outlines represent the nucleus. Scale bars, 10 μm. **g**, Quantification of images in **f**. Imaris was used to identify astrocyte nuclei based on *Aldoc* expression, and the spots feature was used to quantify the number of *Kcnk1* puncta per cell (*n* = 4 mice per group; each data point represents the mean of *Kcnk1*^+^ puncta per *Aldoc*^+^ astrocyte from each mouse; data are mean ± s.e.m.; *P* values by one-way ANOVA followed by Dunnett’s multiple comparison test). **h**, Example confocal images of AQP4 immunofluorescence in mouse prefrontal cortex. Astrocytic endfeet (magenta) are visualized and ensheath the blood vessel. **i**, The polarization index of AQP4 (*n* = 4 (no stimulation), 4 (8 Hz), 4 (40 Hz) and 3 (80 Hz) 6-month-old 5XFAD mice; data are mean ± s.e.m.; *P* values by one-way ANOVA followed by Dunnett’s multiple comparison test). Source Data

**Extended Data Fig. 8:**
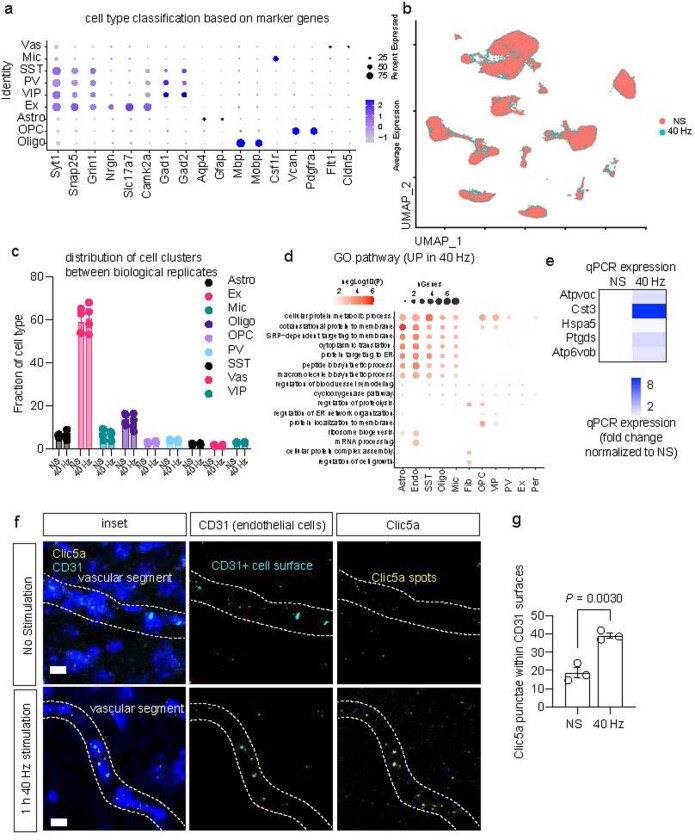
snRNA-seq cell type annotation, analysis, and quality control. a. Marker genes used for cell type annotation. Cells were classified as excitatory neurons (Ex), parvalbumin interneurons (PV), somatostatin interneurons (SST), and vasoactive intestinal peptide interneurons (VIP), microglia (Mic), astrocytes (Astro), oligodendrocyte precursor cells (OPCs), oligodendrocytes (Oligo), and vascular cells (Vas). Dot size indicates percent expressed, and dot color indicates average gene expression level. b. Distribution of cell types based on stimulation condition between no stimulation (NS) and 40 Hz stimulation (40 Hz). c. Distribution of cell cluster numbers based on biological replicate between no stimulation (NS) and 40 Hz stimulation (40 Hz) (*n* = 4 biological replicates per group, with each replicate consisting of three 6-month 5XFAD mouse cortex; data is presented as the mean ± s.e.m.; unpaired two-tailed t-test was used for statistical analysis). d. GO analysis of DEGs following snRNA-seq. The test for GO term enrichment was Fisher exact test. e. qPCR was conducted for genes upregulated in at least 3 cell types. A heatmap of expression change is presented, with fold difference calculated using the 2^−ΔΔCt^ method types (*n* = 5 6-month-old 5XFAD mice per group, performed with two technical duplicates). f. Example confocal z-stack projections of RNA in situ hybridization (RNAscope) in the prefrontal cortex (PFC) of 6-month-old 5XFAD mice with probes to detect CD31 (cyan, endothelial cell marker, not differentially expressed between groups) and Clic5a (yellow; upregulated in 40 Hz). The dotted line represents a putative vascular segment. Scale bar, 10 μm. g. Quantification of *Clic5a* (yellow spots) in endothelial cells (cyan surface rendering) increased in 40 Hz stimulation (40 Hz) compared to no stimulation (NS) (*n* = 3 6-month-old 5XFAD mice per group; each data point represents the mean number of *Clic5a* puncta from CD31+ endothelial cells from 4 confocal z-stacks per mouse; data is presented as the mean ± s.e.m.; P value was calculated by unpaired two-tailed student’s t-test). Source Data

To gain insights into the molecular mechanisms governing CSF influx following gamma stimulation, we turned to single-nucleus RNA sequencing (snRNA-seq). We used 6-month-old 5XFAD mice exposed to multisensory gamma stimulation for 1 h (control mice had no stimulation), allowed mice to rest for 1 h, and then isolated nuclei from whole cortices for snRNA-seq, pooling cortices from three 6-month-old 5XFAD mice for each of 4 replicates per condition (Fig. 3a). We obtained 61,062 nuclei across 9 major cell types, including excitatory neurons, inhibitory neurons (parvalbumin, somatostatin and VIP interneurons), microglia, astrocytes, oligodendrocytes, oligodendrocyte precursor cells and vascular cells (Fig. 3b and Extended Data Fig. 8a). We further annotated vascular cells as endothelial cells, pericytes, smooth muscle cells and fibroblasts^28^ (Fig. 3c). No significant differences in cell proportions were detected across samples (Extended Data Fig. 8b,c). Multisensory gamma stimulation resulted in 144 downregulated genes and 219 upregulated genes, which were particularly evident in endothelial cells, astrocytes and interneurons (Fig. 3d,e and Extended Data Fig. 8d). We verified our analysis using quantitative PCR (qPCR) for genes that were upregulated across multiple cell types (Extended Data Fig. 8e) and in situ hybridization of the transcript *Clic5a* in *Pecam1*^+^ endothelial cells (Extended Data Fig. 8f,g).

**Extended Data Fig. 9:**
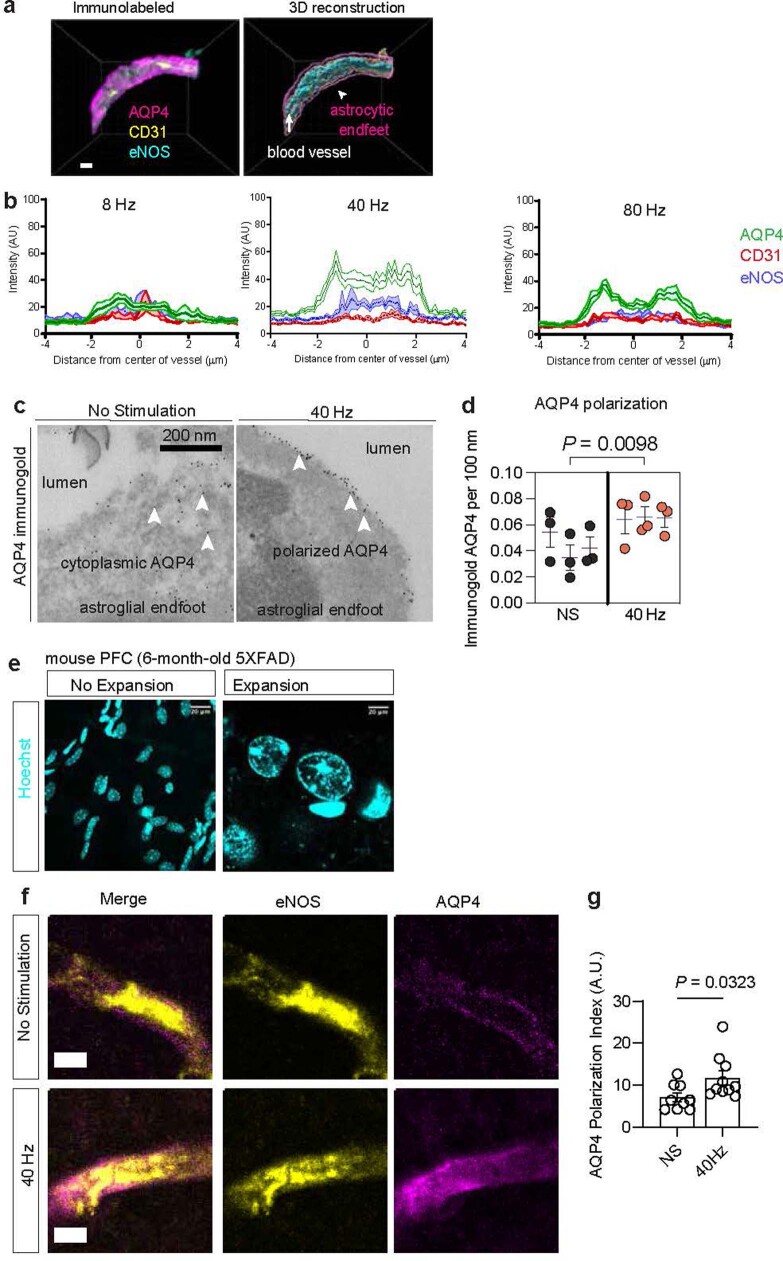
Aquaporin analysis following sensory stimulation. a. Example confocal image of immunohistochemistry of AQP4 in mouse PFC. Astrocytic endfeet (magenta) are visualized and ensheath the blood vessel, visualized based on endothelial nitric oxide synthase (eNOS) and platelet endothelial cell adhesion molecule-1 (PECAM-1/CD31). 3D reconstruction using Imaris reveals astrocytic endfeet ensheath the blood vessel. Similar vascular segments were obtained for all experiments involving AQP4 polarization analyses presented. b. Example analysis of AQP4 polarization and Imaris 3D reconstruction of astrocytic endfeet from the PFC of a 6-month-old 5XFAD mouse. A perpendicular line segment of astrocytic endfeet was drawn in ImageJ, and the signal intensity of AQP4 was plotted. The distribution of the intensity reveals the polarization of AQP4. The polarization index is quantified by dividing the peak signal intensity of the AQP4 line segment normalized to the background intensity. An increase in the ratio between the peak of AQP4 compared to the background signal intensity signifies an increase in AQP4 polarization. Red, CD31 signal. Green, AQP4 signal. Blue, eNOS signal. The polarization of the green AQP4 signal highlights the ensheathment of AQP4 signal around blood vasculature. Shaded areas represent standard error of the mean. c. Example images of transmission electron microscopy (TEM) resolving astrocytic endfeet and AQP4 distribution (dark puncta). 6-month-old mice received one hour of noninvasive multisensory gamma stimulation or no stimulation control, and 40 μm sections of PFC coronal sections were prepared for TEM imaging. d. Quantification of AQP4 distribution via TEM within astrocytic endfeet in 6-month-old 5XFAD mice (n = 3 6-month-old 5XFAD mice per group; data is presented as the mean ± s.e.m. in a nested plot; *P* was calculated by nested two-tailed t-test). e. Validation of Expansion Microscopy using DAPI from mouse cortex. Left = no expansion. Right = post expansion; the experiment was repeated three times. Scale bar, 20 μm. f. Example confocal images of Expansion Microscopy of astrocytic endfeet labeled with AQP4 (magenta), the endothelial cell marker endothelial nitric oxide synthetase (yellow). Scale bar, 5 μm. g. Quantification of aquaporin-4 polarization (*n* = 9 astrocytic endfeet segments for no stimulation and 10 astrocytic endfeet segments for gamma stimulation; data is presented as the mean ± s.e.m.; *P* was calculated by unpaired two-tailed student’s t-test). Source Data

Given the importance of astrocytes to glymphatic function^3,10^, we interrogated astrocytic transcriptional responses to multisensory 40 Hz stimulation. Genes that were upregulated in astrocytes were significantly enriched in membrane protein organization (Extended Data Fig. 8d). To test for the effect of non-40 Hz stimulation effects on astrocytic transcriptional response, we used RNAscope to monitor transcripts of *Kcnk1*, which encodes a highly regulated potassium channel that is thought to be localized to astrocytic endfeet^29^, which are critical for brain fluid transport. We found an increase in the number of *Kcnk1* puncta per *Aldoc*^+^ cell after multisensory 40 Hz stimulation compared with non-40 Hz-stimulated controls in 6-month-old 5XFAD mice (Fig. 3f,g and Supplementary Video 4). Given the astrocytic transcriptional changes related to ion handling relevant for glymphatic function, we tested whether 40 Hz multisensory stimulation modulated AQP4 function. Of note, our snRNA-seq experiment revealed that pericytes upregulate *Lamc1*, which encodes a laminin subunit that is known to interact with dystroglycan, an important component of the anchoring complex responsible for stabilizing AQP4 in the plasma membrane of astrocytic endfeet via syntrophin and dystrophin^30^. Astrocytic gene ontology terms related to the plasticity of astrocyte membrane trafficking following gamma stimulation, including genes associated with structural remodelling (*Rhou* and *Epha5*) and endoplasmic reticulum shaping (*Rtn1*, *Tmcc1* and *Arl6ip1*). AQP4 is dynamically and rapidly trafficked between intracellular vesicles and the astrocytic plasma membrane^31^, and its polarity governs glymphatic function^21^ and amyloid clearance^14,32^. To explore the possibility that gamma stimulation might increase AQP4 polarization along astrocytic endfeet, we exposed 6-month-old 5XFAD mice to 1 h of multisensory gamma stimulation and immunohistochemically evaluated AQP4 using high-resolution confocal microscopy (Fig. 3h and Supplementary Video 5). Polarization analysis along perpendicular segments of AQP4^+^ endfeet^12,21^ (Extended Data Fig. 9a,b) revealed that multisensory 40 Hz stimulation increased AQP4 polarization along astrocytic endfeet in 6-month-old 5XFAD mice compared with non-40 Hz-stimulated controls (Fig. 3i). We confirmed these results using transmission electron microscopy and immunogold-labelled AQP4 (Extended Data Fig. 9c,d) as well as protein retention expansion microscopy to visualize AQP4 in astrocytic endfeet (Extended Data Fig. 9e–g). Collectively, these results reveal that multisensory 40 Hz stimulation promotes AQP4 polarization and suggest that gamma-induced CSF movement into the brain may be facilitated by increased astrocytic AQP4 polarity.

## VIP neurons regulate clearance by gamma

We next explored whether soluble factor(s) released during gamma rhythms act on vascular and glial cells to promote glymphatic clearance of amyloid. Neuropeptides are released in a frequency-dependent manner^33,34^, and are therefore candidate substrates to govern the prolonged cellular effects that we observe following 40 Hz multisensory stimulation. We found that several neuropeptides were transcriptionally upregulated in interneurons, including genes related to somatostatin (*Sst*), neuropeptide Y (*Npy*) and vascular nerve growth factor (*Vgf*). Of note, VIP interneurons upregulated transcripts related to neuropeptide synthesis and secretion—for example, increased expression of *Chgb*, which encodes secretogranin-1, a secretory protein found ubiquitously in the cores of peptide neurotransmitter dense-core secretory vesicles; *Vgf*, which encodes nerve growth factor, a secreted protein and neuropeptide precursor; and *Bsg*, which encodes basigin, a coreceptor for vascular endothelial growth factor. Collectively, these transcriptional results highlight a possible response of neuropeptide signalling following gamma stimulation. To dissect the functional role of neuropeptides in gamma-mediated glymphatic clearance, we began by interrogating VIP interneurons. VIP is a 28-amino-acid peptide that is thought to be released by VIP interneurons during high-frequency stimulation. VIP is associated with attenuation of Alzheimer’s disease pathology^35^, and prostaglandin (which our snRNA-seq and qPCR experiments revealed is upregulated following gamma stimulation), is known to act synergistically with VIP to regulate vascular cells and blood flow. VIP signalling is also involved in several cellular processes that are relevant to glymphatic clearance, including regulation of blood vessel diameter^36^, astrocyte metabolism^37^, aquaporin trafficking^38^, sleep^39^ and circadian rhythms^40^.

**Extended Data Fig. 10:**
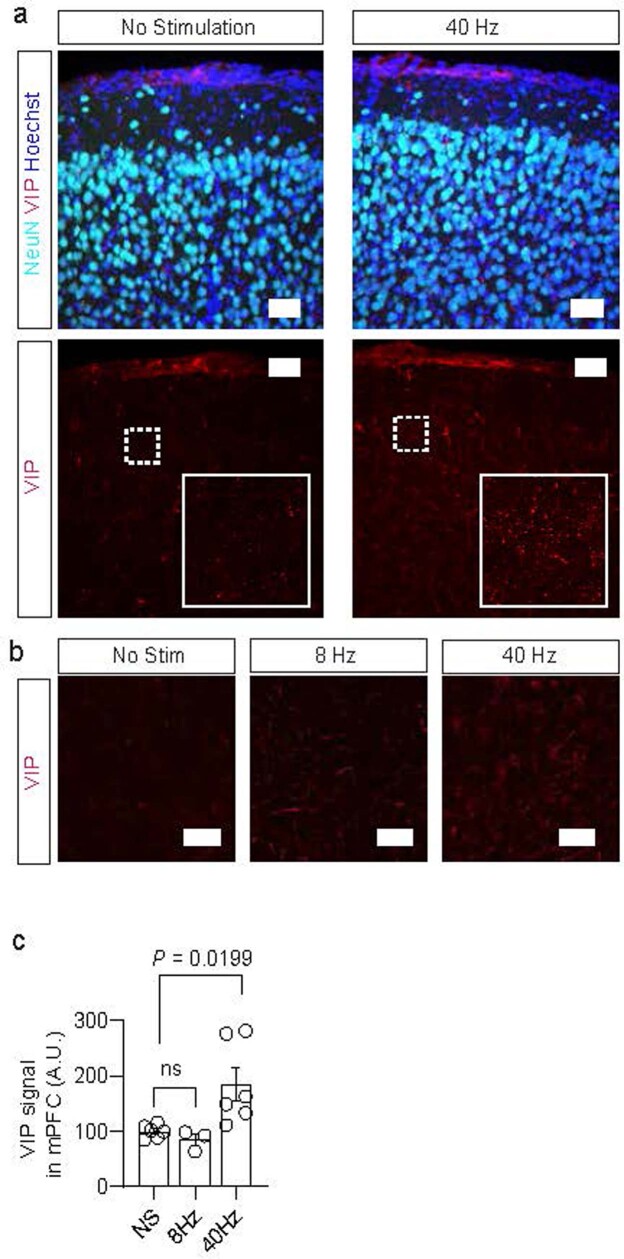
Immunohistochemistry for vasoactive intestinal peptide (VIP) following gamma stimulation. a. Example images of VIP (red) and NeuN (cyan) immunohistochemistry in 6-month-old 5XFAD mouse prefrontal cortex following gamma stimulation or no-stimulation control. Insets reveal increased VIP immunofluorescence in gamma-treated mice. Scale bar, 50 μm. b. Example images of VIP immunohistochemistry in 6-month-old 5XFAD mouse cortex following gamma stimulation or control (red, VIP). Scale bar, 20 μm. c. Quantification of VIP signal intensity (*n* = 5 mice for no stimulation, 3 mice for 8 Hz stimulation, and mice for 40 Hz stimulation; each data point represents the mean from 3 z-stacks of the prefrontal cortex; data is presented as the mean ± s.e.m.; *P was calculated by one-way ANOVA followed by Dunnett’s multiple comparisons test). Source Data

**Extended Data Fig. 11:**
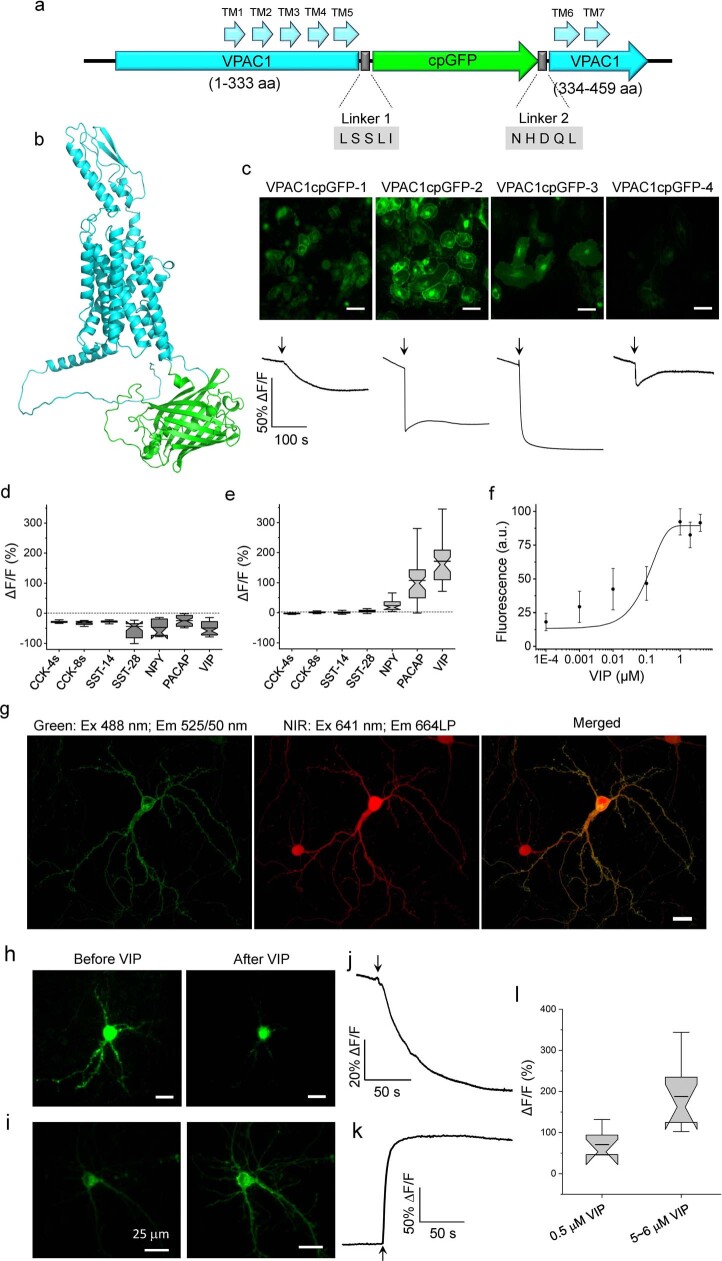
Development and validation of a genetically encoded sensor for vasoactive intestinal peptide (VIP). a. Molecular design of VIP sensor used for in vivo recordings. b. Prediction of the VIP sensor 3D structure using Alphafold v2.1 (computed on Galaxy provided by High-performance computing center at Westlake University). c. Optimization VIP sensor in HeLa cells; (top) representative images of HeLa cells expressing different variants of VIP sensor (n = 2 FOVs from two independent transfections each), (bottom) representative fluorescence traces for each tested variant of VIP sensor (n = 30, 37, 46, and 23 cells from 1, 2, 3, and 2 independent transfections, respectively; arrow indicates time point of VIP administration). d. Fluorescence changes of the VPAC1L sensor expressed in HEK293T cells in response to the indicated compounds applied to the extracellular solution (n > 19 cells from two independent transfections). e. Fluorescence changes of the VIP1.0 sensor expressed in HEK293T cells in response to the indicated compounds applied to the extracellular solution (n > 32 cells from two independent transfections). f. Normalized dose-response curve of the VIP1.0 sensor expressed in HEK cells to VIP (n = 50 cells from two independent transfections). Squares represent the mean, error bars represent the standard deviation. g. Representative image of a cultured primary neuron co-expressing the VIP sensor and miRFP (n = 31 neurons from two independent cultures). h. Representative image of a cultured primary neuron expressing the VIP sensor before and after VIP administration (n = 31 neurons from two independent cultures). i. Representative image of a cultured primary neuron expressing the VIP1.0 sensor before and after VIP administration (n = 10 neurons from four independent cultures). j. Fluorescence trace for neuron shown in h (arrow indicates time point of VIP administration). k. Fluorescence trace for neuron shown in i (arrow indicates time point of VIP administration). l. Fluorescence changes of the VIP1.0 sensor expressed in cultured neurons in response to VIP at the indicated concentrations (n = 6 and 10 neurons from four independent transfections). Box plots with notches: narrow part of notch, median; top and bottom of the notch, 95% confidence interval for the median; top and bottom horizontal lines, 25% and 75% percentiles for the data; whiskers extend 1.5-fold the interquartile range from the 25th and 75th percentiles; horizontal line, mean. Source Data

**Extended Data Fig. 12:**
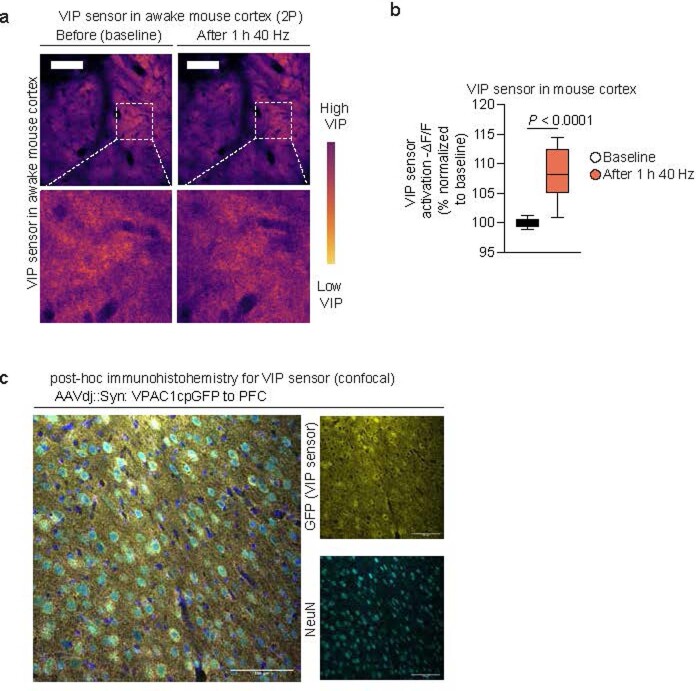
VIP sensor in awake mouse cortex. a. Example 2 P images of VIP sensor (VPAC1cpGFP) expressed in awake mouse cortex visualized through a cranial window centered over prefrontal cortex before and after 1 h of multisensory gamma stimulation. The reduction in signal signifies an increase in VIP sensor activation following 40 Hz treatment; the experiment was repeated twice. Scale bar, 25 μm. b. Quantification of VPAC1cpGFP fluorescence change before and after 40 Hz treatment in 6-month-old 5XFAD mouse cortex via a closed cranial window. The increase in fluorescence change signifies an increase in VIP sensor activation following noninvasive multisensory gamma stimulation (presented as VIP sensor activation, i.e., -ΔF/F). A two-sided Student’s t-test was performed for data analysis (*n* = 15 ROIs from 3 mice imaged before and after gamma stimulation; box plots depict the median, interquartile range, and minimum and maximum; *P < 0.0001 by unpaired two-tailed student’s t-test). c. Immunohistochemistry of VIP sensor in mouse cortex; the experiment was repeated twice. Scale bar, 100 µm. Source Data

To test the hypothesis that neuropeptide signalling is associated with noninvasive multisensory gamma stimulation and glymphatic clearance of amyloid, we performed immunohistochemistry for VIP in the prefrontal cortex of 6-month-old 5XFAD mice and found increased VIP signal in gamma-treated mice (Extended Data Fig. 10a–c). To further interrogate neuropeptide signalling in the context of noninvasive gamma stimulation, we designed a genetically encoded sensor whereby optical readout of changes in neuropeptide concentration was achieved by directly coupling binding-induced conformational changes in the VIP receptor VPAC1 to reduce the fluorescence intensity of circularly permuted green fluorescent protein (cpGFP) (Extended Data Fig. 11a). We inserted cpGFP from the genetically encoded dopamine indicator dLight1.1^41^ using linker sequences (LSSLI-cpGFP-NHDQL) into the third intracellular loop of the VIP receptor VPAC1 (Extended Data Fig. 11a,b). We observed changes in fluorescence intensity following application of vasoactive neuropeptide agonists compared with saline control in HEK293T cells (Extended Data Fig. 11c), and we compared fluorescence changes in our sensor and a similar sensor^42^ to structurally similar peptides (Extended Data Fig. 11d,e) and varying concentrations of VIP (Extended Data Fig. 11f–l and Supplementary Video 6). Next, we broadly labelled frontal mouse cortex using AAVdj::Syn:VPAC1cpGFP. Two-photon imaging through a cranial window in awake mice revealed expression of the sensor (Extended Data Fig. 12a). One hour of gamma multisensory stimulation increased activation of the sensor compared with pre-stimulation levels (Extended Data Fig. 12b), an effect that we confirmed was not owing to photobleaching or repeated imaging, on the basis of quantifying fluorescence intensity in the absence of stimulation via repeated imaging sessions. Given that the sensor responds to structurally similar peptides (Extended Data Fig. 11d,e), we cannot rule out the likely possibility that other vasoactive peptides participate in the effects of multisensory 40 Hz stimulation. Further mining neuropeptide signalling cascades that are recruited during distinct patterns of neuronal activity may provide additional insight into the peptidergic signalling relevant to clearance pathways. Collectively, these results suggest that gamma stimulation involves peptide signalling in the cortex of 5XFAD mice.

**Fig. 4:**
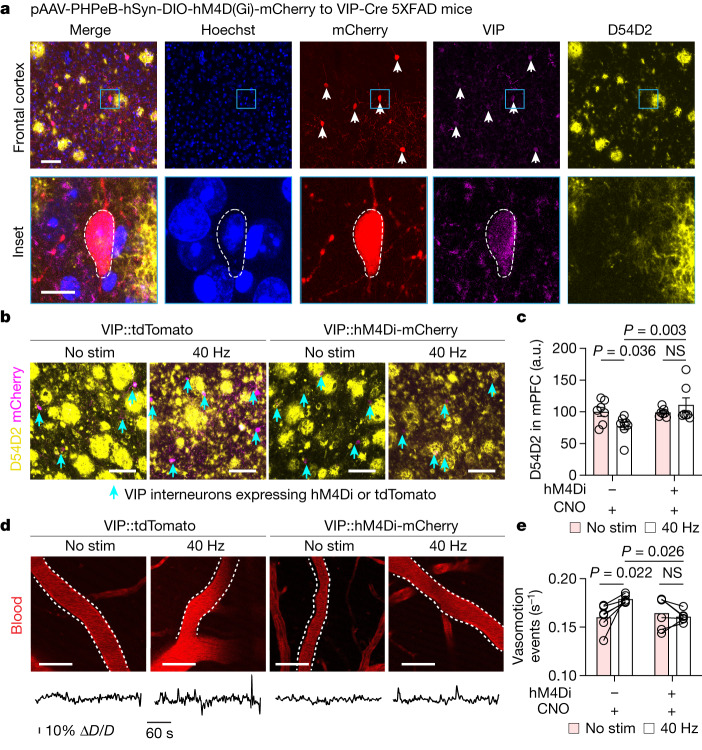
VIP neurons mediate gamma-mediated glymphatic clearance. **a**, Top row, example image of frontal cortex of 6-month-old VIP-Cre 5XFAD mice after receiving PHP.eB.AAV.Syn.DIO-hM4Di-mCherry. Bottom row, magnified view of indicated region in the top row. This experiment was repeated twice. Scale bar: 50 μm (top row) and 10 μm (bottom row). **b**, Example confocal *z*-stack maximum intensity images of medial prefrontal cortex (mPFC) of 6-month-old VIP-Cre 5XFAD mice injected with PHP.eB.AAV.Syn.DIO-hM4Di-mCherry (VIP::hM4Di-mCherry) or control (VIP::tdTomato) and labelled with D54D2 and mCherry (indicated with cyan arrowheads) receiving no stimulation or 40 Hz stimulation. Scale bars, 100 μm. **c**, Quantification of amyloid in experiment represented in **b** (*n* = 7 (no stimulation + tdTomato), 8 (40 Hz + tdTomato), 7 (no stimulation + hM4Di) and 7 (40 Hz + hM4Di) VIP-Cre 5XFAD mice; data are mean ± s.e.m.; *P* values by two-way ANOVA followed by Fisher’s LSD test). **d**, Example images and time series of arterial pulsatility after multisensory stimulation. Scale bars, 50 μm. **e**, Quantification of arterial pulsatility (*n* = 5 VIP-Cre 5XFAD per group; data are mean ± s.e.m.; *P* values by two-way ANOVA followed by Fisher’s LSD test). Source Data

**Extended Data Fig. 13:**
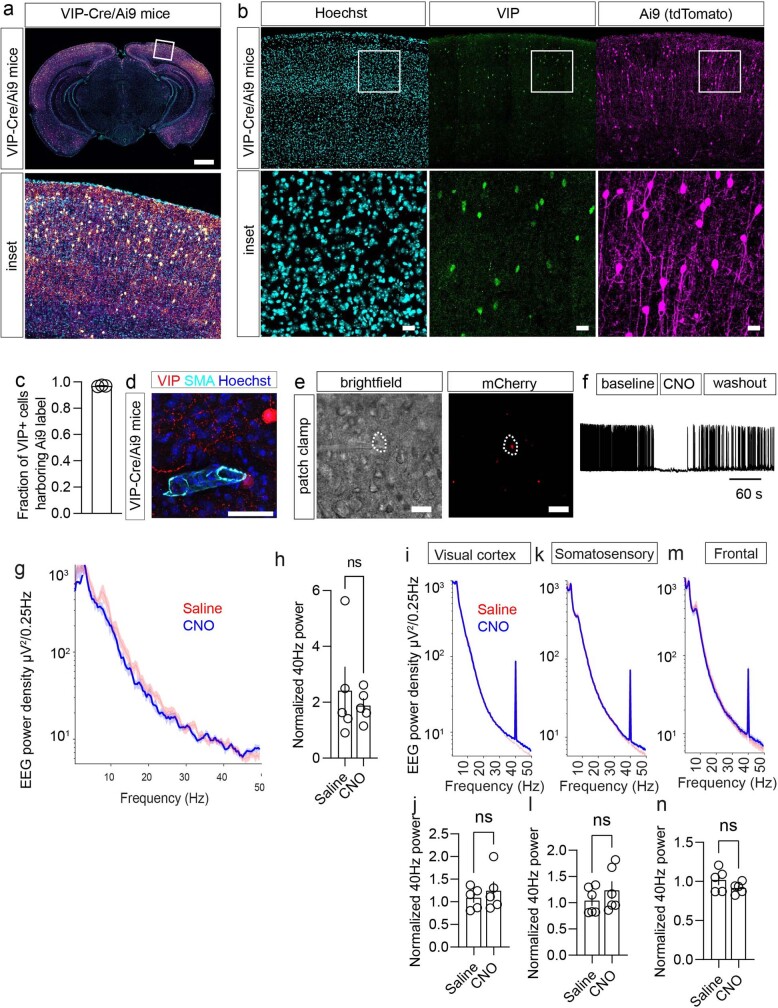
VIP-Cre mouse validation and effects on 40 Hz neuronal activity. a. Example coronal section of VIP-Cre/Ai9 mouse acquired with confocal microscopy. VIP-IRES-Cre mice have Cre recombinase expression directed to VIP-expressing cells by the endogenous promoter/enhancer elements of the vasoactive intestinal polypeptide locus. The experiment was repeated twice. Scale bar, 1000 um. b. Example confocal z-stack maximum intensity projection image of immunohistochemistry for VIP (green) and tdTomato using a primary antibody against mCherry (magenta), with Hoechst (cyan) from a VIP-Cre/Ai9 mouse cortex. Scale bar, 20 um. c. Quantification of cells expressing both Ai9 and VIP (n = 3 mice; data is presented as the mean ± s.e.m.). d. Example of VIP neuronal processes (red, VIP-Cre tdTomato) adjacent to arterial smooth muscle (cyan, labeled with SMA-22). Scale bar, 50 µm. e. Example brightfield (left) and fluorescent (right) images of the patched neuron expressing mCherry. A giga-ohm seal was achieved for recording. VIP-Cre/5XFAD mice were retro-orbitally injected with PHP.eB-AAV-DIO-hM4Di-mCherry. Virus was allowed to express for ~4 weeks. Scale bar, 50 um. f. Whole cell current clamp recording of VIP neurons expressing Gi-coupled DREADDs from prefrontal cortex. Represented sweep shows bath application of CNO (20 uM) following washout. g. Poewr density in the frontal EEG showing the effect of VIP chemogenetic inhibition on baseline state. VIP-Cre/5XFAD mice received PHPeb-AAV-DIO-hM4Di-mCherry, EEG implants were placed, then recordings were obtained such that mice received either CNO or saline prior to recording during multisensory 40 Hz stimulation (*n* = 5 6-month-old 5XFAD mice; data is present as the mean; shaded region represents the s.e.m.). h. Quantification of normalized 40 Hz power (*n* = 5 6-month old VIP-Cre/5XFAD mice; data is presented as mean ± s.e.m.; paired two-tailed t-test was used for analysis). i. Mean EEG power density measured in the visual cortex of VIP-cre during 1 h of multisensory 40 Hz stimulation performed after the injection of either saline or CNO (*n* = 5 6-month old VIP-Cre/5XFAD mice; mean across mice; shaded area = SEM). j. Normalized 40 Hz EEG power following saline or CNO in visual cortex (*n* = 5 6-month old VIP-Cre/5XFAD mice; data is presented as the mean ± s.e.m. paired two-tailed t-test was used for analysis). k. Mean EEG power density measured in the somatosensory cortex of VIP-cre during 1 h of multisensory 40 Hz stimulation performed after the injection of either saline or CNO. (Mean across mice; shaded area = SEM). l. Normalized 40 Hz EEG power following saline or CNO in somatosensory cortex (*n* = 5 6-month old VIP-Cre/5XFAD mice data is presented as the mean ± s.e.m.; paired two-tailed t-test was used for analysis). m. Mean EEG power density measured in the frontal cortex of VIP-cre during 1 h of multisensory 40 Hz stimulation performed after the injection of either saline or CNO (mean across mice; shaded area= SEM). n. Normalized 40 Hz EEG power following saline or CNO in frontal cortex (*n* = 5 6-month old VIP-Cre/5XFAD mice; data is presented as the mean ± s.e.m.; paired two-tailed t-test was used for analysis). Source Data

To causally determine whether VIP interneurons regulate amyloid clearance during gamma stimulation, we chemogenetically inhibited VIP interneurons using PHP.eb.AAV::Syn:DIO-hM4Di-mCherry in VIP-Cre 5XFAD transgenic mice. We confirmed that VIP-Cre mice label cortical VIP interneurons with high fidelity and specificity^43^ (Extended Data Fig. 13a–c). As expected^44^, we found that PHP.eB labelled VIP neurons throughout the cortex of VIP-Cre 5XFAD mice (Fig. 4a). The hM4Di ligand clozapine-*N*-oxide (CNO) reduced neuronal activity of VIP neurons expressing hM4Di (Extended Data Fig. 13e,f) and—notably—did not substantially affect baseline 40 Hz power or the 40 Hz neural response to multisensory 40 Hz stimulation, as measured by in vivo electrophysiology recordings in 6-month-old VIP-Cre 5XFAD mice (Extended Data Fig. 13g–n). Chemogenetically inhibiting VIP neurons before gamma stimulation attenuated amyloid clearance (Fig. 4b,c) and arterial pulsatility compared with non-hM4Di CNO-only control in 6-month-old VIP-Cre 5XFAD mice (Fig. 4d,e). Furthermore, inhibiting VIP neurons attenuated the effect of multisensory 40 Hz stimulation on AQP4 polarization (Supplementary Fig. 1), and pharmacologically modulating VIP further highlighted a role of VIP in regulating AQP4 in a reduced blood–brain barrier culture (Supplementary Fig. 2), consistent with other biological systems indicating a role of vasoactive compounds in regulating aquaporin trafficking and mobilization. These results suggest that multisensory 40 Hz stimulation promotes peptide signalling to induce glymphatic clearance in part through changes in arterial pulsations (Supplementary Fig. 3).

## Discussion

Following the observation that optogenetic gamma stimulation can attenuate amyloid burden^5^, we found that sensory gamma stimulation promotes 40 Hz neural activity in multiple brain regions including the prefrontal cortex^6,7,16^. Sensory gamma stimulation also attenuates Alzheimer’s disease-related pathology^5^, ameliorates neurodegeneration^7^ and morphologically transforms astrocytes^6^ and microglia^5–7^ in models of Alzheimer’s disease. These observations have been replicated and extended^45–48^, but the cellular processes by which sensory stimulation attenuates amyloid load in the brain have remained incompletely resolved. In this study, we investigated the role of glymphatic clearance during multisensory gamma stimulation. Given that the glymphatic system clears parenchymal amyloid^3^ and is regulated by neural rhythms^18^, we hypothesized that glymphatic clearance might account for amyloid reduction by sensory gamma stimulation. Gamma rhythms may be relevant to aspects of glymphatic clearance as gamma may be involved in aspects of sleep^49,50^ and neuronal synchrony. Moreover, the envelope of gamma rhythms entrains arterial pulsatility^20^, which is thought to govern CSF influx. Leveraging a variety of imaging modalities to monitor multiple aspects of the glymphatic pathway, as well as genetic and molecular interventions, we found that gamma stimulation promoted the influx of CSF into the cortex and the efflux of ISF, which was associated with increased arterial pulsatility, increased AQP4 polarization, and dilation of meningeal lymphatic vessels. Our findings support observations indicating that brain rhythms govern CSF dynamics^1,18,51^. Our experiments also suggest that aspects of gamma rhythms, such as arterial pulsatility^20^ and peptide signalling, may contribute to CSF dynamics. The long-lasting nature of peptide signalling may explain in part why increased vasomotion persists following the end of stimulation.

Glymphatic influx is attributed to the sleeping state. A recent report suggests that noninvasive sensory stimulation modulates CSF outflow in awake humans^52^, potentially suggesting that CSF and glymphatic pathways can be modulated in the awake brain. Our findings suggest that multisensory 40 Hz stimulation promotes amyloid clearance and activates glymphatic pathways. Future work may disentangle how cellular pathways regulating glymphatic systems in various arousal states are modulated.

The meningeal lymphatic drainage network, arterial-driven CSF movement and neuronal network dysfunction contribute to amyloid pathology in Alzheimer’s disease^14^. Our observations suggest that brain rhythms contribute to arterial regulation of CSF clearance and meningeal lymphatic drainage. We found that multisensory gamma stimulation increased arterial pulsatility in a VIP interneuron-dependent manner. Consistent with prior results highlighting electrophysiological and haemodynamic coupling of CSF dynamics^9,51^, our experiments highlight an interconnected relationship between glial, neuronal and vascular cells in the regulation of CSF dynamics. Transcriptional responses to gamma stimulation—which we highlighted using both snRNA-seq and in situ hybridization—further indicate that transcriptional responses related to ion channels may have a critical role in linking neural oscillations with glial and vascular responses governing fluid transport. Our results also suggest that vasoactive signals from neurons act on glial and vascular cells during neural oscillations to drive CSF clearance. Future work further defining factors linking brain rhythms and vasoactive clearance may enhance the therapeutic potential of recruiting glymphatic transport for the treatment of neurodegenerative disorders associated with the accumulation of amyloid peptides and potentially other pathogenic extracellular proteins.

## Methods

### Mice

All animal experiments were conducted in accordance with National Institutes of Health (NIH) guidelines and were overseen by and adherent to the rules set forth by the Massachusetts Institute of Technology Institutional Animal Care and Use Committee. All of the animal holding rooms were maintained within temperature (18–26 °C) and humidity ranges (30–70%) described in the ILAR Guide for the Care and Use of Laboratory Animals (1996). Mice were housed in groups no larger than five on a standard 12 h–12 h light–dark cycle (lights on at 07:00; all experiments were performed during the light cycle). All efforts were made to keep animal usage to a minimum, and male and female mice were used. 5XFAD (Tg 6799) breeding pairs were acquired from the Mutant Mouse Resource and Research Center (MMRRC) (Jax 034848) and crossed with C57BL/6 J mice to generate offspring for this study. For experiments involving genetic manipulations of VIP^+^ interneurons, Vip^tm1(cre)Zjh^/J (Jax 010908) were crossed to 5XFAD to generate VIP-Cre 5XFAD heterozygous mice. To determine the specificity and fidelity of the Cre expression in VIP-Cre mice, we used B6.Cg-Gt(ROSA)26Sor^tm9(CAG-tdTomato)Hze^/J (Jax 007909) to indelibly label VIP interneurons with tdTomato and to generate Ai9/VIP-Cre 5XFAD triple transgenic mice. Since circadian rhythms and brain state are known to regulate glymphatic flux and AQP4 polarization^21^, all experimental groups were evaluated at consistent levels in the circadian cycle (~2–6 h after lights on).

### Noninvasive multisensory stimulation

Multisensory stimulation was performed as described previously^6^. In brief, mice were moved from the vivarium and held in a quiet room. Following 1 h of habituation to the room, individual mice were placed in separate chambers. The chamber was illuminated by a light-emitting diode programmed to either 8 Hz (125 ms light on, 125 ms light off), 40 Hz (12.5 ms light on, 12.5 ms light off, 60 W), or 80 Hz. Speakers (AYL, AC-48073) were placed above the chambers and programmed to present a 10 kHz tone that was 1 ms in duration and delivered at 60 decibels tones at 8 Hz or tones at 40 Hz. The LED and speakers were programmed via a microcontroller (Teensy) such that the sensory input was delivered simultaneously (that is, stimulus pulses of each modality were aligned to the onset of each pulse).

### Tissue collection and processing for immunohistochemistry

Following sensory stimulation or control, mice were given a lethal dose of anaesthetic (isoflurane overdose) then transcardially perfused with PBS (pH 7.4) with heparin (10 U ml^−1^, Sigma H3149) followed by PBS with 4% paraformaldehyde (PFA) (Electron Microscopy Sciences 15710). Whole mounts of the dural meninges were prepared as described^11,24^. Following perfusion, skull caps were removed, then placed in 4% PFA at 4 °C for 12 h. The dural meninges (dura mater and arachnoid) were peeled from the skull cap under a dissecting microscope using Dumont forceps (Fine Science Tools) then placed in a 24-well plate (VWR 10861-558) with PBS for immunohistochemistry. Deep cervical lymph nodes were dissected, fixed in 4% PFA for 16 h, then gently cleaned under a dissecting microscope to gently remove non-lymph node surrounding tissue. Lymph nodes were then dehydrated in 30% sucrose until the lymph nodes sank, embedded in OCT (Tissue-Tek), then frozen at −80 °C, then cut at 40 µm in a cryostat and mounted on SuperFrost slides. Immunohistochemistry was then conducted on slide-mounted tissue sections. Lymph node sections mounted on slides and treated for immunohistochemistry (described below). Brains were kept in 4% PFA for 18–24 h, then washed in PBS, the cut using a vibratome into 40 μm thick sections. Coronal brain sections were kept in PBS at 4 °C until preparation for immunohistochemistry.

### Immunohistochemistry

Lymph nodes, coronal brain sections, and meninges were treated for immunohistochemistry using the following protocol. First, tissue was washed with PBS for 10 min, permeabilized with 0.3% Triton X-100 in PBS for 10 min, underwent blocking (5% normal donkey serum and 0.3% Triton X-100 in PBS) for 1 h at room temperature, and immunostained with the primary antibodies in blocking solution overnight. Following three 5-min washes with blocking buffer, we added secondary antibodies in blocking buffer for 2 h at room temperature, then washed with PBS five times for 5 min each. A list of antibodies are provided in Supplementary Table 1 (Supplementary Information). On the penultimate wash we used 1:1,000 Hoechst (Thermo Fisher Scientific, H3570). Tissue was mounted on SuperFrost slides and sealed with Prolong Gold mounting medium (Thermo Fisher Scientific, P36930).

### Confocal microscopy

We used a Zeiss confocal 710, 880 or 900 for confocal microscopy. The same microscope was used for each imaging experiment, and identical imaging settings were used for all settings acquired by the blinded investigator. For quantification of amyloid in lymph node, we imaged regions of lymph node in draining regions based on CD31/LYVE1 staining and imaged at 425.10 µm^2^ (1.204 pixels per µm), at 11 µm *z-*stacks at 2-µm step sizes. For quantification of amyloid in the prefrontal cortex, the region to be imaged was selected based on Hoechst reference and comparison with the mouse brain atlas, then we imaged a region of 319.45 µm^2^ (3.2055 pixels per µm) using a 30 µm *z*-stack imaged at 1-µm step sizes. To ensure consistency and unbiased imaging by the blinded investigator, we used the Hoechst channel to set the upper and lower boundaries of each *z-*stack. Zeiss ZEN Blue (v3.3.89) (Carl Zeiss Microscopy) was used for image acquisition. For data analysis, Fiji image processing software (v1.54) (NIH) and Imaris (v9.1) (Oxford Instruments) were used.

### Pharmacology

To modulate AQP4 function in mice, we used TGN020 (TargetMol T5102), administered 30 min prior to sensory stimulation (100 mg kg^−1^, intraperitoneal injection). We used this dose based on prior literature suggesting a modulation of CSF distribution^13^. For experiments involving VIP, we used HSDAVFTDNYTRLRKQMAVKKYLNSILN (19113, Bachem); for VIP receptor agonists, we used acetyl-(d-Phe2,Lys15,Arg16,Leu27)-VIP (1–7)-GRF (8–27) (202463-00-1, Bachem), [Lys15, Arg16, Leu27]-VIP (1–7)-GRF (8–27) (064-24, Phoenix Pharmaceuticals); for VIP receptor antagonists, we used [d-*p*-Cl-Phe6,Leu17]-VIP (3054, Tocris). Peptides were aliquoted and stored at −20 °C.

### Generation of AAV5-GFAP-EGFP-shAqp4 and AAV5-GFAP-EGFP-shLacZ

To selectively reduce AQP4 in astrocytes, we synthesized AAV delivering eGFP followed by miR30-based shRNA^26^ targeting mouse *Aqp4* under the astrocyte-specific GFAP promoter (AAV-EGFP-shAqp4). To broadly reduce AQP4 levels, we designed three target sequences for AQP4 knockdown. As a control, we designed AAV carrying eGFP with *lacZ* shRNA (AAV-EGFP-shLacZ). Oligonucleotides containing shAqp4 or shLacZ within miR30 backbone were synthesized (IDT), annealed, and cloned into pAAV.GFAP.eGFP.WPRE.hGH (Addgene plasmid #105549) using the NheI site. All constructs were assembled using standard cloning methods and confirmed by DNA sequencing. Plasmids expressing miR30-based shAqp4 or shLacZ was packaged into AAV5 (Janelia Viral Core). The sequence of oligonucleotides can be found in Supplementary Table 4.

### Cranial windows

Anaesthesia was induced using isoflurane (induction, 3%; maintenance, 1–2%), ophthalmic ointment (Puralube Vet Ointment, Dechra) was applied to the eyes to prevent corneal drying, and metacam (1 mg kg^−1^ intraperitoneal injection) and buprenorphine (0.05 mg kg^−1^, subcutaneous injection) were administered as analgesics. Mice were placed in a stereotactic frame (Kopf Instruments) and a heating pad was used to maintain body temperature. Scalp fur was trimmed and treated with three alternating swabs of betadine and 70% ethanol. A small circular section of skin (~1 cm in diameter) was excised using surgical scissors (Fine Science Tools). The periosteum was bluntly dissected away and bupivacaine (0.05 ml, 5 mg ml^−1^) was topically applied as a topical analgesic. A circular titanium headplate was attached to the skull using dental cement (C&B Metabond, Parkell), centred around prefrontal cortex (1.7 mm anterior to bregma, centred over the midline). Under a continuous gentle flow of PBS (137 mM NaCl, 27 mM KCl, 10 mM phosphate buffer), a ~ 4-mm circular section of the skull, slightly larger than the window, was removed using a 0.5-mm burr (Fine Science Tools) and a high-speed hand dental drill, taking great care not to compress brain tissue or damage dural tissue. Sugi swabs (John Weiss & Son) were used to absorb trace bleeding. A 3-mm glass coverslip (Warner Instruments) was gently placed over the brain. Veterinary adhesive (Vetbond, Fisher Scientific) was used to form a seal between the coverslip and the skull. A layer of Metabond was then applied for added durability. The mouse was then placed in a cage, half-on and half-off of a 37 °C heating pad, until it regained sternal recumbency. Metacam (1 mg kg^−1^ intraperitoneal injection) was administered as an analgesic 24 h after surgery, and as needed thereafter. Mice were allowed 3–4 weeks of recovery before imaging.

### Intracisterna magna cannulation

We followed previous reports in order to perform intracisterna magna cannulation^53^. Mice were anaesthetized with isoflurane (3% induction, 1% maintenance), ophthalmic ointment (Puralube Vet Ointment, Dechra) was applied to the eyes, and the head and neck were shaved and sterilized with povidone-iodine (Dynarex) and 70% ethanol. 1 mg ml^−1^ of bupivacaine was injected subcutaneously at the incision site and buprenorphine (0.05 mg kg^−1^, subcutaneous injection) was administered for preemptive analgesics. The mouse was fixed in the stereotaxic frame (Knopf) by the zygomatic arch, and the head was titled to form a 120° angle with the body. The occipital crest was identified, the overlying skin (~1 cm) cut, and sterile forceps were used to pull apart the superficial connective tissue and neck muscles in an anterior-to-posterior direction to expose the cisterna magna, where the cerebellum and medulla were visible behind the translucent dural membrane. A cotton swab (Sugi) was used to dry the dural membrane and a 30 G needle prepared prior to surgery fixed with PE10 tubing (Polyethylene Tubing 0.024” OD x 0.011” ID, BD Intramedic) and filled with fresh artificial CSF (ACSF) (126 mM NaCl, 2.5 mM KCl, 1.25 mM NaH_2_PO_4_, 2 mM MgSO_4_, 2 mM CaCl_2_, 10 mM glucose, 26 mM NaHCO_3_) was carefully inserted through the dural membrane, carefully avoiding damage to the cerebellum and medulla. Trace CSF leak was dried using sterile cotton swabs (Sugi), and cyanoacrylate glue (Loctite) was used to secure the cannula into the dural membrane and glue accelerator was applied to cure the glue. The needle was then secured in place using dental cement (Parkell) and a handheld cauterizer (Fine Science Tools) was used to seal the tubing. The mouse was then placed in a cage, half-on and half-off of a 37° heating pad, until it regained sternal recumbency. Following recovery from cannulation, CSF tracer infusion and awake stimulation was conducted.

### Awake in vivo two-photon imaging

Mice were head-fixed to a custom titanium head fork using no. 0-80 screws. The head-fixed mouse was positioned over a 3D printed running wheel covered in waterproof neoprene foam. Mice quickly learned to run or quietly rest (motionlessly) while in a head-fixed position. We habituated mice to gentle handling and this head-fixed position for 3 days prior to imaging experiments to avoid motion artefacts during the experiment. Prior to imaging, and while the mouse was head-fixed, the cranial window was gently cleaned using a cotton-tipped swab and a small ~1 ml dollop of Aquasonic Clear Ultrasound Transmission Gel (Parker) was placed over the cranial window. Two-photon microscopy images were acquired using an Olympus FVMPE-RS microscope. A low magnification image was acquired to facilitate returning to the same imaging site over time, and high-resolution, high numerical aperture imaging was used to acquire experimental data.

### Two-photon imaging of CSF tracer

Mice had received a cranial window (3 weeks prior) and intracisterna magna implant (~3 h prior) and habituated to awake head-fixed imaging. We prepared fluorescent CSF tracer (fluorescein-conjugated dextran, 3 kD, Invitrogen D3306), formulated to a 0.5% solution in ACSF (126 mM NaCl, 2.5 mM KCl, 1.25 mM NaH_2_PO_4_, 2 mM MgSO_4_, 2 mM CaCl_2_, 10 mM glucose, 26 mM NaHCO_3_). We infused 10 µl of tracer via a cisterna cannula into awake mice at a rate of 1 μl min^−1^ for 10 min with a syringe pump (WPI), a rate we chose based on prior reports^11,22^, sealed the tube using a handheld cauterizer (Fine Science Tools, 18010-00), and placed mice in a chamber for 1 h of noninvasive multisensory stimulation or control. Following 1 h of stimulation, mice were head-fixed and positioned under the objective. To visualize tracer movement from the cisternal compartments into the brain parenchyma, we used a Spectra-Physics InsightX3 DeepSee laser tuned to 920 nm to visualize CSF tracer (labelled by fluorescein dextran) and blood vessels (labelled via retroorbital injection of Texas Red–dextran 70 kD injected prior to the experiment). Fluorescence was collected using a 25×, 1.05 numerical aperture water immersion objective with a 2-mm working distance (Olympus), and signal was detected through gallium arsenide phosphide photomultiplier tubes using the Fluoview acquisition software (Olympus). We simultaneously acquired images in the red channel (bandpass filter 575–645 nm) to visualize vascular arbors and in the green channel (bandpass filter 495–540 nm) for CSF tracer. We imaged *z*-stacks using a galvano scanner (*z*-stacks were 200 µm from the cortical surface, imaged at 2-µm step sizes; the imaging rate was set to 2.0 µs per pixel for the 512 × 512 pixel region, covering ~509.117 µm^2^). Three areas were imaged per mouse. Tracer influx was quantified by a blinded investigator using ImageJ and Imaris, and an average fluorescence intensity was calculated between *z*-stacks and normalized to non-treated mice.

### Ex vivo fluorescence imaging of CSF tracer

Fluorescent CSF tracer (OVA-647; 45 kDa; O34784, Invitrogen) was formulated to a 0.5% solution in ACSF (126 mM NaCl, 2.5 mM KCl, 1.25 mM NaH_2_PO_4_, 2 mM MgSO_4_, 2 mM CaCl_2_, 10 mM glucose, 26 mM NaHCO_3_). We infused 10 µl of tracer via a cisterna cannula into awake mice at a rate of 1 μl min^−1^ for 10 min with a syringe pump (WPI), a rate we chose based on prior reports suggesting that this method only maintains intracranial pressure following infusion^11,22^. To maintain intracranial pressure following infusion, we sealed the tube using a handheld cauterizer (Fine Science Tools, 18010-00). We then placed mice in a chamber for 1 h of noninvasive multisensory stimulation or control and 1 h of recovery. Since death is associated with the collapse of paravascular space and non-physiological influx of CSF^23^, we sought to avoid this potential confound entirely, so mice were euthanized within 60 s of the end of the experiment via isoflurane overdose, decapitated, and brain was fixed overnight by immersion in 4% paraformaldehyde in PBS at 4 °C with gentle rotation. To visualize tracer movement from the cisternal compartments into the brain parenchyma, we sliced brain sections at 100 µm using a vibratome (Leica) and imaged fluorescence on a Zeiss 880 confocal microscope (425.1 µm^2^ imaging region; 1.2044 pixels per µm). Tracer influx was quantified by a blinded investigator using ImageJ. The cerebral cortex in each slice was manually outlined, and the mean fluorescence intensity within the cortical regions of interest was measured. An average of fluorescence intensity was calculated between six slices for a single mouse, resulting in a single biological replicate. Equivalent coronal brain slices were used for all biological replicates.

### Two-photon imaging of arteriole pulsation

To image arterial pulsation, we labelled vasculature using Texas Red–dextran 70 kD via retroorbital injection prior to the experiment. Mice previously fixed with a cranial window had been habituated to head fixation under the two-photon imaging apparatus for awake imaging. We used a Spectra-Physics InsightX3 DeepSee laser tuned to 920 nm. Fluorescence was collected using a 25×, 1.05 numerical aperture water immersion objective with a 2-mm working distance (Olympus), and signal was detected through gallium arsenide phosphide photomultiplier tubes using the Fluoview acquisition software (Olympus). We acquired images in the red channel (bandpass filter 575–645 nm) for blood plasma. In a subset of experiments, we also acquired images in the green channel (bandpass filter 495–540 nm) for microglia, and movement in the green channel was used for motion artefact detection and were easily detected. We used a resonance scanner to acquire time series of arterial pulsatility in awake mice. A single recording was 328.90 s and covered an area of 160.7 µm^2^ at a rate of 0.067 ms per pixel and 0.127 ms per line; in total, 5,000 frames were recorded at an imaging rate of 65.779 ms per frame. We validated the absence of motion artefact in our analysis based on the absence of vessel change in venous segments obtained in the same imaging areas as the arterial segments, as well as by using the soma of microglia in CX3CR1 5XFAD mice. To avoid subtle *xy* changes in motion, we used the phase correlation rigid registration method implemented in suite2p, using the microglia channel to align the vascular channel. To quantify arterial pulsatility, we used a perpendicular segment of the artery binarized using ImageJ, and the diameter segment was quantified using Python: first, a savgol filter (window size 7, polynomial order 5) was applied to the vasomotion trace, and peaks were identified using find_peaks.

### Two-photon microscopy interstitial efflux assay by laser ablation

To image ISF efflux by laser ablation, we recorded vascular segments spanning an area of 169.706 µm^2^. For baseline imaging, we imaged at a rate of 65.779 ms per frame, 0.067 ms per pixel, 0.127 ms per line for 5,000 frames at a rate of 65.779 ms per frame. We imaged vascular beds using Spectra-Physics InsightX3 DeepSee laser tuned to 920 nm (IR laser power set at 2.22 W, and imaged using ~3.5–4.5% transmissivity). To induce ablation, we used a second two-photon laser (Mai Tai DeeSee) laser tuned to 800 nm (IR laser power at 2.79 W and transmissivity at 20–30%). Next, we induced an ellipsis region of interest for stimulation, drawn along a vascular segment approximately 3 µm in diameter. We induced stimulation using the following settings: 80 μs per pixel, 3.20 μs per line, for a total of 100 ms. Following successful ablation, a bolus of dextran was removed, and we used the InsightX3 to continue imaging to monitor the efflux and diffusion of the extravasted dextran (imaging for 328.90 s, covering an area of 160.7 µm^2^ at 65.779 ms per frame for 5,000 frames). In pilot experiments to validate the reperfusion of blood vessels following focal ablation, we used line scans of blood vessels perfused and volumetric scans of the surrounding vascular area, using single line scans in the central lumen of along 15 µm for a capillary segment. Space-time scans were acquired using one-way galvano scanning, and the line speed was 1.989 μm per pixel for 5.7 s (5,000 frames). We performed this assay in three areas per mouse following gamma stimulation, and quantified the rate of efflux by quantifying the ratio of the extravasted dextran signal intensity at the peak of the extravasation and the end of the diffusion period, using identical distances between vascular segments between both treatment groups.

### EMG and EEG data acquisition and analysis

Electroencephalogram (EEG) and electromyography (EMG) implants were installed in 6-month-old 5XFAD mice under isoflurane anaesthesia as described^54^. For analysis of sleep architecture based on EEG and EMG recordings, all mice were included. All mice implanted for electrophysiological recordings were housed individually in open cages before surgery and in individually ventilated cages during a recovery period of about 1 week after surgery. For sleep recordings, mice were transferred to separate custom-made Plexiglas cages (20.3 × 32 × 35 cm), which were placed in sound-attenuated and light-controlled Faraday chambers (Campden Instruments), with each chamber fitting two cages. Mice were allowed free access to food pellets and water at all times and underwent daily health inspection. After an acclimatization period of at least 3 days, during which mice were habituated to the tethered recording conditions, a period of continuous recording starting at light onset was performed on a designated baseline day. On the subsequent day, all mice received either no stimulation, 40 Hz noninvasive multisensory stimulation, or 8 Hz noninvasive multisensory stimulation conditions (see ‘Noninvasive multisensory stimulation’) and were recorded for the entire 1-h stimulation period and the entire 1 h of post-stimulation. Recordings between groups were conducted at the same time of day because circadian rhythms affect glymphatic clearance^21^. EMG and EEG data were acquired using Synapse (Tucker–Davis Technologies) and continuously recorded, filtered between 0.1 and 100 Hz, and stored at a sampling rate of 305 Hz. EEG and EMG signals were resampled at a sampling rate of 256 Hz using custom code in MATLAB (MathWorks, v2017a). Sirenia Sleep Pro (v2.2.1, Pinacle Technology) was used for sleep scoring. EEG and EMG recordings were partitioned into epochs of 4 s. Vigilance states were assigned manually to each recording epoch based on visual inspection of the frontal and occipital EEG derivations in conjunction with the EMG. Epochs with recording artefacts due to gross movements, chewing or external electrostatic noise were assigned to the respective vigilance state but not included in the electrophysiological analysis. Overall, 18.8% ± 3.5% of wake, 0.7% ± 0.4% of NREM and 0.9% ± 0.4% of REM epochs contained artefactual EEG signals across all mice included in the EEG spectral analysis, with no significant difference between stimulation conditions. EEG and LFP power spectra were computed using a fast Fourier transform routine (Hanning window) with a 0.25-Hz resolution.

### Behaviour

The novel object recognition task consisted of a habituation phase followed by training and testing, as used in our lab previously^6^. Mice were habituated in an open field testing box for 10 min on 3 consecutive days. On the fourth day, 2 identical wooden blocks (Premium wooden building blocks set, Cubbie Lee) were placed in the chamber, and mice were allowed to explore the objects for 10 min, then the mice were returned to their home cage. Twenty-four hours later in the test phase, one of the wooden blocks was switched to a novel wooden block with a different shape, and the time spent exploring the familiar and new objects was measured for 10 min. Discrimination index was calculated as time spent to explore the new object divided by the sum of time spent to explore both old and new object by a recognition index. EthoVision (XT 14) (Noldus) was used for behaviour tracking.

### Expansion microscopy

Forty-micrometre coronal brain sections fixed in 4% PFA were expanded according to protein expansion protocols. In brief, after immunolabelling with anti-AQP4 and anti-eNOS, samples were treated with AcX overnight, gelled for 2 h at 37 °C, and digested with proteinase K overnight. After expansion, samples were imaged using a glass bottom plate (Cellvis, P06-1.5H-N) and imaged using an inverted Zeiss LSM 710 confocal microscope.

### Electron microscopy

Perfused brains were in 4% PFA in PBS and post fixed in 4% PFA in PB overnight at 4 °C. Sections were then washed in 0.02 M glycine for 15 min. Brains were cut at 40 µm using a vibratome, then permeabilized in 0.1% Triton X-100, blocked in 1% BSA, and incubated with rabbit-anti-AQP4 overnight at 4 °C. Preparation was completed at the Harvard Electron Microscopy Core. For epon embedding, 0.5% osmium was added for 30 min, washed in water, then dehydrated using ethanol. Propyleneoxide was used and infiltrated in propyleneoxide and TAAB Epon overnight. Sections were flat embedded between two sheets of Aclar in fresh TAAB Epon, then polymerized at 60 °C for 48 h. Ultrathin sections (~60 nm) were cut on a Reichert Ultracut-S microtome, picked up on to copper grids stained with 0.2% lead citrate and examined in a JEOL 1200EX Transmission electron microscope. Images were recorded with an AMT 2k CCD camera.

### AQP4 polarization analysis

We used established methods to quantify AQP4 polarization^12,21^. We found that AQP4 labelled astrocytic endfeet that ensheathed blood vessels as well as surrounding parenchyma. AQP4 segments were selected on confocal *z*-stack projections, then marked cross-sectionally using the line plot tool in ImageJ to include AQP4 signal from vascular endfeet and from the surrounding parenchyma. The ratio of AQP4 signal from endfeet to parenchyma fluorescence intensity ratio was used as a measure of AQP4 polarization.

### Isolation of single nuclei for snRNA-seq

The protocol for the isolation of nuclei from frozen post-mortem brain tissue was adapted from a previous study^55^. All procedures were carried out on ice. Following 1 h of gamma stimulation or control and 1 h of rest, cortices were dissected and snap frozen in liquid nitrogen and stored at −80 °C. Then, 3 mouse cortices were pooled per sample (4 samples per condition) and homogenized in 1 ml homogenization buffer (320 mM sucrose, 5 mM CaCl_2_, 3 mM Mg(CH_3_COO)_2_, 10 mM Tris HCl pH 7.8, 0.1 mM EDTA pH 8.0, 0.1% IGEPAL CA-630, 1 mM β-mercaptoethanol, and 0.4 U µl^−1^ recombinant RNase inhibitor (Clontech)) using a Wheaton Dounce tissue grinder (15 strokes with the tight pestle). The homogenized tissue was filtered through a 40-μm cell strainer, mixed with an equal volume of working solution (50% OptiPrep density gradient medium (Sigma-Aldrich), 5 mM CaCl_2_, 3 mM Mg(CH_3_COO)_2_, 10 mM Tris HCl pH 7.8, 0.1 mM EDTA pH 8.0, and 1 mM β-mercaptoethanol) and loaded on top of an OptiPrep density gradient (29% OptiPrep solution (29% OptiPrep density gradient medium,134 mM sucrose, 5 mM CaCl_2_, 3 mM Mg(CH_3_COO)_2_, 10 mM Tris HCl pH 7.8, 0.1 mM EDTA pH 8.0, 1 mM β-mercaptoethanol, 0.04% IGEPAL CA-630, and 0.17 U µl^−1^ recombinant RNase inhibitor)) on top of 35% OptiPrep solution (35% OptiPrep density gradient medium, 96 mM sucrose, 5 mM CaCl_2_, 3 mM Mg(CH_3_COO)_2_, 10 mM Tris HCl pH 7.8, 0.1 mM EDTA pH 8.0, 1 mM β-mercaptoethanol, 0.03% IGEPAL CA-630, and 0.12 U µl^−1^ recombinant RNase inhibitor). The nuclei were separated by ultracentrifugation using an SW32 rotor (5 min, 10,000*g*, 4 °C). Nuclei were collected from the 29%–35% interphase, washed with PBS containing 0.04% BSA, centrifuged at 300*g* for 3 min (4 °C) and washed with 1 ml of PBS containing 1% BSA. The nuclei were counted and diluted to a concentration of 1,000 nuclei per μl in PBS containing 1% BSA. Libraries were prepared using the Chromium Single Cell 3′ Reagent Kits v.3.1 (Dual Index) according to the manufacturer’s protocol (10X Genomics). The generated scRNA-seq libraries were sequenced using NextSeq 500/550 High Output (150 cycles).

### Analysis of droplet-based snRNA-seq data

Raw reads were aligned to the mouse genome and the gene counts were estimated by CellRanger software (v3.0) (10X Genomics)^56^. Seurat (v4.0.3) was used for downstream analysis^57^. Cells with more than 500 protein-coding genes with detected unique molecular identifiers from protein-coding genes were selected for further analysis. We also use the ratio of mitochondrial genes to measure the quality of cells (cells with higher than 5% were removed). We used DoubletFinder to remove the potential doublets from snRNA-seq data. The top 2,000 highly variable genes were used for principal component analysis. The first 30 principal components were used for non-linear dimensionality reduction (UMAP) for visualization. FindMarkers function in Seurat was used to identify marker genes for each cluster and each cell type, and DEGs between mice receiving gamma stimulation or no stimulation control. For DEG analysis, the cut-off used in the function FindMarkers in Seurat was: min.pct: 0.25, only test genes that are detected in a minimum fraction of min.pct cells in either of the two populations; logfc.threshold: 0.25. Enrichr was used to perform the Gene Ontology enrichment analysis^58^ with *P* value < 0.05 as a cut-off. Negative log_10_-transformed *P* value was used for visualization by heat map with the selected representative terms based on the diverse functional categories. A list of DEGs are available in Supplementary Table 3 (Supplementary Information)

### RNA extraction and qPCR with reverse traancription

Following 1 h of gamma stimulation or control and 1 h of rest, cortices were dissected and snap frozen in liquid nitrogen and stored at −80 °C. Total RNA was extracted using TRIzol (Invitrogen) according to the manufacturer’s instructions. Reverse transcription of total RNA was carried out using RNA to cDNA EcoDry Premix (Clontech) according to the manufacturer’s protocol. qPCR was performed using a Bio-Rad CFX-96 quantitative thermocycler and SsoFast EvaGreen Supermix (Bio-Rad). Relative changes in gene expression were determined using the 2^−ΔΔ*C*t^ method. Primer sequences used for qPCR can be found in Supplementary Table 2.

### RNA in situ hybridization

We used RNAscope for fluorescence in situ hybridization following the manufacturer’s protocol. The probes we used are listed in the appropriate figure legends. Tissue was prepared as in the section above describing tissue preparation with the following deviation. Following overnight fixation at 4 °C in 4% PFA in PBS, brains were cryopreserved using 30% sucrose and cut at 40 µm using a cryostat (Leica). Coronal brain sections were preserved at −80 °C until the RNAscope experiment was conducted.

### Peptide sensor design

We used a sequence analogous to another G-protein-coupled-receptor-based sensor^41^. We replaced the third intracellular loop of the VPAC1 module with a cpGFP module from the genetically encoded calcium indicator GCaMP6 using linker sequences (LSSLI-cpGFP-NHDQL). The linker sequences to the VIP sensor were designed using SnapGene. To generate AAV, we used Janelia Virus Core. For imaging VIP sensor in HeLa cells and mouse neuronal culture, we used wide-field fluorescence imaging using epifluorescence inverted microscope (Eclipse Ti-E, Nikon) equipped with a Photometrics QuantEM 512SC camera and a 75 W Nikon xenon lamp or a Zyla5.5 sCMOS camera (Andor) and a SPECTRA X light engine (Lumencor). NIS-Elements Advanced Research (Nikon) was used for automated microscope and camera control. Cells were imaged with a 60× NA1.49 oil or 20× NA0.75 air objective lenses (Nikon) at room temperature. For dual-colour imaging with miRFP, NIR (650/60 nm excitation and 720/50 nm emission) and green (490/15 nm excitation and 525/50 nm emission) filter sets were rotated into the emission light path. The GRABVIP1.0 sensor was provided by Y. Li. HEK293T cells (Invitrogen) cultured in Dulbecco’s modified Eagle’s medium (Gibco) with 10% fetal bovine serum (FBS, YEASEN Biotech) were seeded on 15 mm cover glasses (Wuxi NEST Biotech) coated with Matrigel (Millipore) and incubated at 37 °C with 5% CO_2_ for 24 h before transfection. Cells were transfected with liposomal methods according to the manufacturer’s protocol (Hieff Trans, YEASEN Biotech). HEK293T cells were imaged 24 h post-transfection by an inverted wide-field Nikon Eclipse Ti2 microscope equipped with a SPECTRA III light engine (Lumencor) and an Orca Flash4.0v3 camera (Hamamatsu), controlled by NIS-Elements AR software and using a 20× 0.75 NA objective lens. Cells were imaged in the Tyrode buffer (150 mM NaCl, 4 mM KCl, 2 mM MgCl_2_, 2 mM CaCl_2_, 10 mM glucose and 10 mM HEPES at pH 7.35). The stock solutions of neuropeptides including CCK-4s (lot no. ab141328, Abcam), SST-14 (lot no. SP-50401-1, Alpha Diagnostic), SST-28 (lot no. SP-52221-1, Alpha Diagnostic), NPY (lot no. ab120208-500 µg, Abcam), PACAP (lot no. HY-P0176A, MedChemExpress), VIP (lot no. B6079-1, Tocris) were dissolved in water, except for CCK-8s (lot no. ab120208-1 mg, Abcam) dissolving in 0.1% NH_4_OH. The working concentration of corresponding neuropeptides was 1 µM in Tyrode buffer. These neuropeptides were administrated to transfected cells via manual addition or replacing the medium with the diluted buffer using custom build perfusion system. Hippocampal neurons were prepared from postnatal day 0–1 C57BL/6 J mouse pups as described. In brief, the hippocampi were dissected in HBSS and digested with 0.25% Trypsin (Yeason) at 37 °C for 12 min. After digestion, the hippocampi were washed three times with plating culture medium (90% advanced MEM + 10% FBS) and then aspirated to dissociate the neurons. The dissociated neurons were plated at a density of 80,000 per 12-mm glass coverslip coated with Matrigel (Corning 356234) in 24-well plate. The next day, the culture medium was half replaced with NeuroBasal Medium supplemented with 1% GlutaMAX and 2% B27. AraC (0.002 mM, Sigma) was added when glia density reached 50–70% confluence. At DIV5-6, neurons were transfected with pAAV-Syn-GRABVIP1.0 or plasmid (1 µg per well) using a commercially available calcium phosphate transfection kit (Life Technologies). At DIV 12–15, fluorescence imaging was performed on an inverted wide-field Nikon Eclipse Ti2 microscope equipped with a SPECTRA III light engine and Orca Flash4.0v3 camera (Hamamatsu), using a 20×, 0.75 NA objective lens. Neurons were incubated in the extracellular solution containing: 150 mM NaCl, 4 mM KCl, 2 mM MgCl_2_, 2 mM CaCl_2_, 10 mM glucose and 10 mM HEPES at pH 7.35. VIP stock solution were diluted with extracellular solution and applied manually using pipette.

### Retroorbital injection for AAV.PHP.EB injections

Mice were anaesthetized by intraperitoneal injection with ketamine-xylazine. The virus was diluted in 100 μl sterile saline and administered in the sinus behind the eye. Following the injection, Puralube was administered and mice were kept at 37 °C until they regained sternal recumbency. Virus was allowed to express for at least 3 weeks.

### Slice preparation and electrophysiological recordings

Six-month-old VIP-Cre 5XFAD mice previously injected with PHPeB-AAV-Syn-DIO-hM4Di-mCherry were deeply anaesthetized with sodium pentobarbital (200 mg kg^−1^, intraperitoneal injection) and then were decapitated. Brains were quickly removed and placed in an oxygenated ice-cold cutting solution containing (in mM): 2.5 KCl, 1.25 NaH_2_PO_4_•H_2_O, 20 HEPES, 2 thiourea, 5 sodium ascorbate, 3 sodium pyruvate, 92 *N*-methyl-d-glucamine, 30 NaHCO_3_, 25 d-glucose, 0.5 CaCl_2_•2H_2_O and 10 MgSO_4_•7H_2_O. Brain slices (180 μm, coronal section) were made using a Leica VT1000S vibratome (Leica Biosystems). Brain slices were incubated in oxygenated cutting solution at 34 °C for 20 min to recover. After recovery, slices were transferred into oxygenated ACSF at room temperature (24 °C) for recording. ACSF solution contains (in mM): 125 NaCl, 2.5 KCl, 1.2 NaH_2_PO_4_, 1.2 MgCl_2_.6H_2_O, 2.4 CaCl_2_•2H_2_O, 26 NaHCO_3_ and 11 d-glucose. A single slice was transferred into a recording chamber and continually superfused with oxygenated ACSF. Cells were visualized using infrared differential interference contrast (IR-DIC) imaging on an Olympus BX-50WI microscope. Action potentials were recorded at 32 °C using the whole-cell current clamp configuration of a patch-clamp amplifier (Multiclamp 700B; Molecular Devices). Action potentials were obtained by a gap-free acquisition mode using Clampex software (Molecular Devices). Signals were filtered at 1 kHz using the amplifier’s four-pole, low-pass Bessel filter, digitized at 10 kHz with a Digidata 1550B interface (Molecular Devices) and stored on a personal computer. Pipette solution contained (in mM) 121 KCl, 4 MgCl_2_•6H_2_O, 11 EGTA, 1 CaCl_2_•2H_2_O, 10 HEPES, 0.2 GTP, and 4 ATP. CNO was applied via bath perfusion.

### iBBB culture

The in vitro blood–brain barrier (iBBB) cultures were created and maintained as described^59^. The iBBB consisted of a co-culture of human astrocytes, endothelial cells, and pericytes co-encapsulated in hydrogel and cultured for two weeks prior to analysis (additional details are available in Supplementary Information) Following iBBB differentiation and culture, VIP receptor agonist was added and 24 h later, cultures were fixed using 4% PFA and imaged using immunohistochemistry using antibodies against human CD31 (also known as PECAM-1) (sheep, R&D systems, AF806) and AQP4 (rabbit, Thermo Fisher, PA5-53234).

### Software

The following software was used to collect the data in this study: Olympus Fluoview (FV31-S, 2.3.1.163) (Olympus); Zeiss ZEN Blue (v3.3.89) (Carl Zeiss Microscopy); EthoVision (XT 14) (Noldus). The following software was used to analyse the data in this study: Fiji image processing software (v1.54) (NIH); Prism (v9.2) (Graph Pad); Python (v3.9); CellRanger (v3.0) (10X Genomics); Seurat (v4.0.3); Imaris (v9.1) (Oxford Instruments).

### Reporting summary

Further information on research design is available in the Nature Portfolio Reporting Summary linked to this article.

## Online content

Any methods, additional references, Nature Portfolio reporting summaries, source data, extended data, supplementary information, acknowledgements, peer review information; details of author contributions and competing interests; and statements of data and code availability are available at 10.1038/s41586-024-07132-6.

### Supplementary information

Supplementary Information Supplementary Methods, Supplementary Figs 1–3 and Supplementary Tables 1–2.

Reporting Summary

Supplementary Table 3 DEGs from snRNA-seq. This Excel file describes the differentially expressed genes from mouse 5XFAD cortex following sensory stimulation or control. For DEG analysis, the cut-off used in the function FindMarkers in Seurat was: min.pct: 0.25, only test genes that are detected in a minimum fraction of min.pct cells in either of the two populations; logfc.threshold: 0.25.

Supplementary Table 4 Oligonucleotide sequences used to target AQP4.

Supplementary Video 1 Two-photon microscopy z-stacks in 6-month-old 5XFAD mouse prefrontal cortex following no stimulation or 1 hour of noninvasive multisensory gamma stimulation revealing CSF tracer (FITC–dextran 3 kD). Cisterna magna-infused CSF tracer (cyan) is labelled in the frontal cortex, and blood vessels (magenta) were labelled with dextran. Volume rendering was performed in Imaris.

Supplementary Video 2 Arterial pulsatility in 6-month-old 5XFAD mouse prefrontal cortex imaged through a cranial window using two-photon microscopy and labelled using Texas Red–dextran 70kD. The video depicts change in diameter over time for each vascular segment. Y axis depicts percent change in diameter over baseline diameter; X axis depicts time (minutes); red dot depicts segment of diameter quantification according to the time frame showed in the image.

Supplementary Video 3 Lymphatic vessel volumes in 6-month-old 5XFAD mouse dural meninges imaged using super resolution AiryScan confocal microscopy and visualized in 3D renderings using Imaris. Lymphatic vessels are visualized using Lyve1. Volume renderings were achieved using the Imaris Surfaces feature.

Supplementary Video 4 RNA in situ hybridization in 6-month-old 5XFAD mouse prefrontal cortex for *Kcnk1* in *Aldoc+* cells imaged using confocal microscopy and quantified using 3D renderings and automated spot detection in Imaris. Aldoc is visualized in yellow, Kcnk1 is visualized in cyan, and DAPI is visualized in blue. Volume renderings were achieved using the Imaris Surfaces feature, and spots were achieved using Imaris Spots detection.

Supplementary Video 5 AQP4 immunohistochemistry (magenta) and polarization analysis in 6-month-old 5XFAD mouse prefrontal cortex and visualized in 3D renderings using Imaris. Example images show AQP4 (magenta) and DAPI (blue) visualized in frontal cortex. High-resolution imaging reveals AQP4 (magenta) around vascular segment labelled with CD31 (cyan) and eNOS (yellow). Volume renderings were achieved in Imaris Surface detection.

Supplementary Video 6 VIP sensor expressed in mouse culture and visualized before and after administration of VIP receptor agonists. VIP sensor activation signal (green) is quantified over time following bath application of VIP receptor antagonist.

### Source data

Source Data Fig. 1

Source Data Fig. 2

Source Data Fig. 3

Source Data Fig. 4

Source Data Extended Data Fig. 1

Source Data Extended Data Fig. 2

Source Data Extended Data Fig. 3

Source Data Extended Data Fig. 4

Source Data Extended Data Fig. 5

Source Data Extended Data Fig. 6

Source Data Extended Data Fig. 7

Source Data Extended Data Fig. 8

Source Data Extended Data Fig. 9

Source Data Extended Data Fig. 10

Source Data Extended Data Fig. 11

Source Data Extended Data Fig. 12

Source Data Extended Data Fig. 13

## Data Availability

Sequencing data is available at the Gene Expression Omnibus (GEO) under accession number GSE249644. All data necessary for the conclusions of this study are presented in the manuscript. Source data are provided with this paper.

## Code availability

The code used for the vasomotion analysis can be found at 10.5281/zenodo.10476268. The code used for the snRNA-seq analysis can be found at 10.5281/zenodo.10480275.
