# Supplementary material for: Multisensory gamma stimulation promotes glymphatic clearance of amyloid
Source: Nature. 2024 Feb 28;627(8002):149–56. doi: 10.1038/s41586-024-07132-6 (PMC10917684; doi:10.1038/s41586-024-07132-6)
Supplement: Supplementary file 1 — Supplementary Methods, Supplementary Figs 1–3 and Supplementary Tables 1–2. [file 41586_2024_7132_MOESM1_ESM.pdf]

nature portfolio

https://doi.org/10.1038/s41586-024-07132-6

Supplementary information

Multisensory gamma stimulation promotes glymphatic clearance of amyloid

In the format provided by the authors and unedited

nature portfolio

https://doi.org/10.1038/s41586-024-07132-6

Supplementary information

Multisensory gamma stimulation promotes glymphatic clearance of amyloid

In the format provided by the authors and unedited

Supplementary Methods

iBBB culture. iBBB cultures were created and maintained as in Blanchard et al 2020[1]. The iBBB consisted of a co-culture of human astrocytes, endothelial cells, and pericytes. co-encapsulated in hydrogel and cultured for two weeks prior to analysis. These iBBB cultures were maintained in astrocyte medium supplemented with 50ng/mL VegF-A for two weeks prior to VIP agonist treatment. Following iBBB differentiation and culture, a VIP agonist was added and 24 hour later, cultures were fixed using 4% PFA and imaged using immunohistochemistry (described in Immunohistochemistry). Antibodies used: Human CD31/PECAM-1 (sheep, R&D systems, AF806); Aquaporin-4 (rabbit, ThermoFisher, PA5-53234). All human ESCs and human iPSCs were maintained in feeder-free conditions in mTeSR1 medium (Stem Cell Technologies) on Matrigel-coated plates (BD Biosciences). The iPSC lines were generated by the Picower Institute for Learning and Memory iPSC Facility iPSCs were passaged at 60–80% confluence using 0.5 mM EDTA solution for 5 min and reseeding 1:6 onto Matrigel-coated plates. For brain endothelial cell differentiation, human ESCs/iPSCs were disassociated to single cells via Accutase and reseeded at 20 x 10^3 cm^{-2} onto Matrigel-coated plates in mTeSR1 supplemented with 10 μM Y27632 (Stem Cell Technologies). The next day, medium was changed to DeSR1 medium (DMEM/F12 with GlutaMAX (Life Technologies) supplemented with 0.1 mM β-mercaptoethanol, 1× MEM-NEAA, 1× penicillin-streptomycin) supplemented with 6 μM CHIR99021 (R&D Systems), and 10ng/ml BMP4 (R&D systems). On day 3, the medium was changed to DeSR2 (DMEM/F12 with GlutaMAX (Life Technologies) supplemented with 0.1 mM β-mercaptoethanol, 1× MEM-NEAA, 1× penicillin-streptomycin and B-27 (Invitrogen)). On day 5, the medium was changed to hECSR1 (Human Endothelial SFM (Thermo Fisher Scientific), B-27, and 1x penicillin-streptomycin) supplemented with 10ng/mL VegF-A (peprotech) and 2uM forskolin (R&D systems). On day 8, cells were split 1:5 using accutase and reseeded with hECSR1 supplemented with 10 μM Y27632 and 10ng/mL VegF-A on Matrigel coated plates. BECs were then cultured in hECSR1 medium supplemented with VegF-A and used within 1 week for experiments. For pericyte differentiation, iPSCs were disassociated to single cells via Accutase, reseeded onto Matrigel-coated plates at 40,000 cells cm^2 in mTeSR1 medium supplemented with 10 μM Y27632. On day 1, medium was changed to N2B27 medium (1:1 DMEM/F12 with GlutaMAX and Neurobasal Medium (Life Technologies) supplemented with B-27, N-2 and penicillin-streptomycin) with 25 ng ml^{-1} BMP4 (Thermo Fisher Scientific, PHC9531) and 8 μM CHIR99021. On days 4 and 5, medium was changed to N2B27, supplemented with 10 ng ml^{-1} PDGF-BB (Peprotech, 100-14B) and 2 ng ml^{-1} Activin A (R&D Systems, 338-AC-010). Pericytes were then maintained in N2B27 medium until they were co-cultured. NPCs were differentiated using dual SMAD inhibition and bFGF supplementation. For astrocytes, NPCs were cultured with Neurobasal NPC medium (DMEM/F12 + GlutaMAX, Neurobasal Medium, N-2 Supplement, B-27 Supplement, 5 ml of GlutaMAX, 10 ml of NEAA and 10 ml of penicillin-streptomycin) supplemented with bFGF (20 ng ml^{-1}). Astrocyte differentiation was induced using astrocyte medium (Sciencell, 1801). Astrocyte medium was changed every other day and cells were passaged at a 1:3 split at 90% confluence. Following cell type differentiation, BECs, pericytes, and astrocytes were co-encapsulated in hydrogel (TrueGel3D, Millipore Sigma) supplemented with a cell-degradable crosslinker (Millipore Sigma) at a ratio of 5:1:1 and seeded at a volume of 20ul per well of an 8-chambered cell culture slide (Mattek).

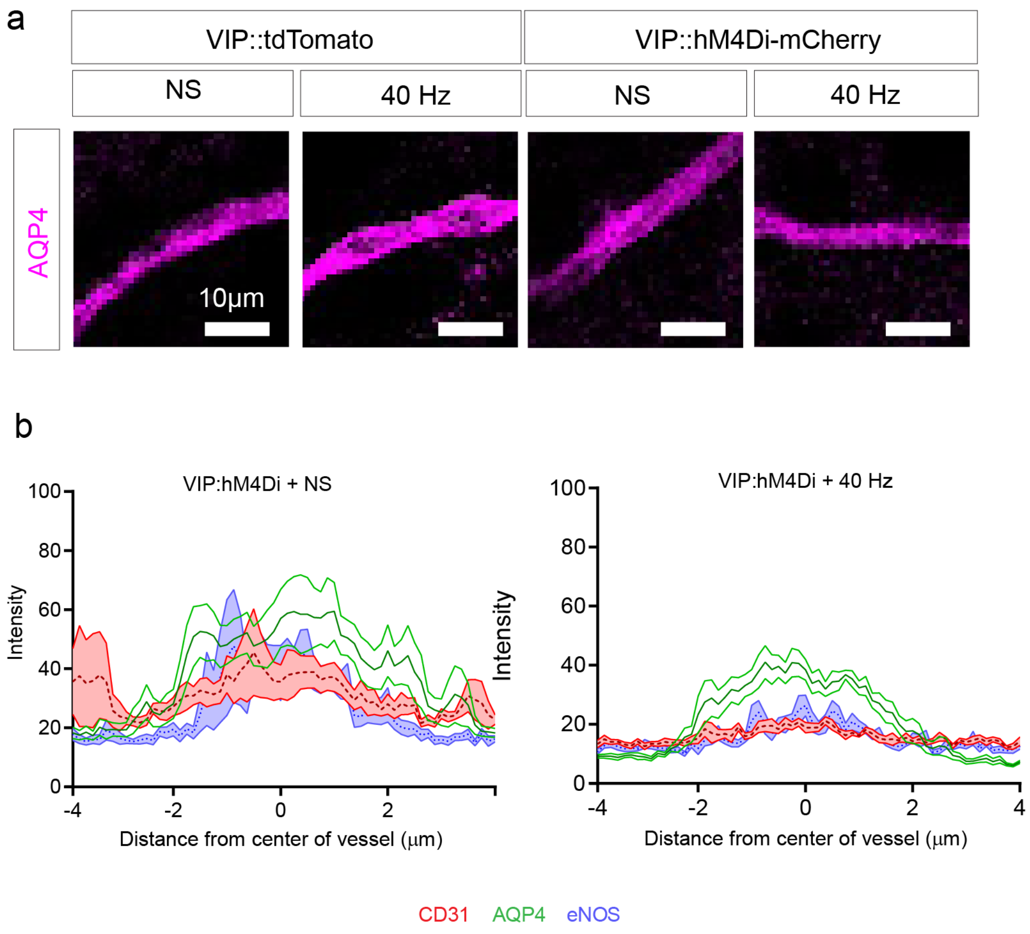

Fig S1. Effect of VIP interneuron chemogenetic inhibition on AQP4 polarization.

A. Example confocal images of AQP4 polarization
B. Example quantification of AQP4 polarization analysis. Line represents the mean and shaded areas represent standard error of the mean.

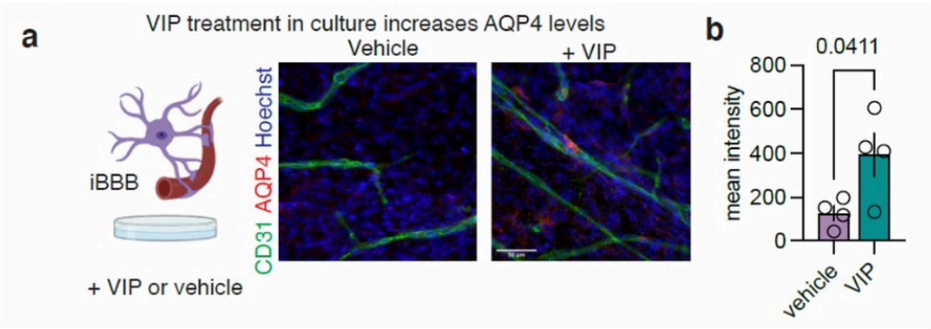

Fig S2. Effect of VIP in an in vitro blood brain barrier cell culture model.

a. Schematic and example images. VIP was added to the iBBB, which included pericytes, endothelial cells, and astrocytes.
b. Quantification of AQP4 signal intensity (N= 4 iBBB cultures per condition; student's t-test; data is presented as the mean ± s.e.m.; unpaired two-tailed student's t-test was used for statistical analysis).

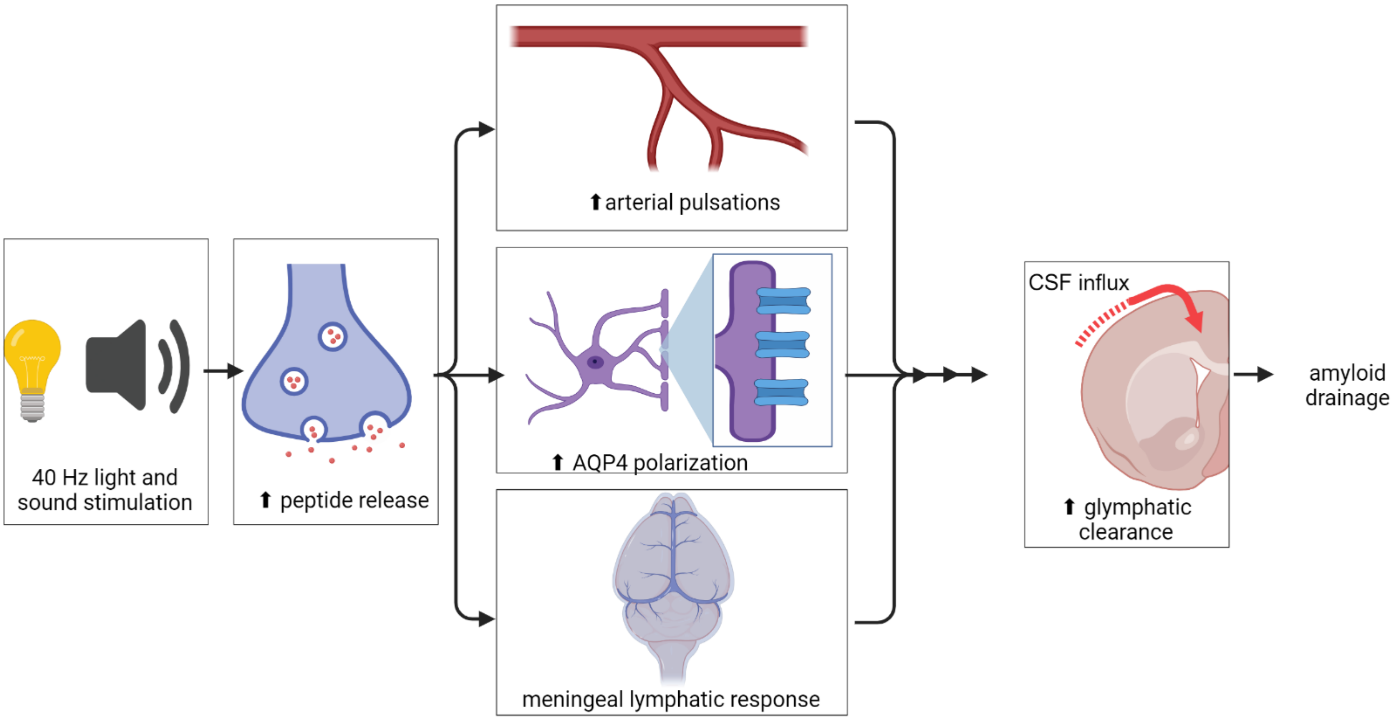

Fig S3. Schematic of glymphatic recruitment by noninvasive sensory gamma stimulation. First, noninvasive audio-visual gamma stimulation promotes 40 Hz rhythms in the brain. Next, neuropeptide release increases following 40 Hz stimulation. Several cellular signaling cascades respond to peptide signaling, including enhanced arterial pulsatility, aquaporin polarization, and meningeal lymphatic response. Collectively, changes in arterial pulsations facilitate CSF influx, and amyloid drainage.

Supplementary References

Supplementary References

1. Blanchard, J. W. *et al.* Reconstruction of the human blood–brain barrier in vitro reveals a pathogenic mechanism of APOE4 in pericytes. *Nat. Med.* **26**, 952–963 (2020).

Supplementary Table 1. Antibodies used for immunohistochemistry

Reagent	Source	Catalog
Anti-Aquaporin4	Thermo Fisher Scientific	PA5-53234
Anti-CD31	Abcam	ab24590
Anti-Connexin 43	Thermo Fisher Scientific	710700
Anti-Cre Recombinase	Millipore Sigma	MAB3120
Anti-eNOS	Abcam	ab76198
Anti-GFAP (2.2B10)	Thermo Fisher Scientific	13-0300
Anti-Ly-6A/E Clone E13-161.7	BD Bioscience	553333
Anti-LYVE1	Abcam	ab14917
Anti-Mlc1	Thermo Scientific	PA5-64327
anti-mouse LYVE-1	Angiobio	11-034
Anti-PECAM-1	Sigma-Aldrich	MAB1398Z
Anti-Syntrophin	Abcam	ab11425
Anti-VIP	ImmunoStar	20077
Anti-VIP Antibody (H-6)	Santa Cruz Biotechnology	sc-25347
Anti-VPAC1 (extracellular)	Alomone Labs	AVR-001
Anti-β-Amyloid (D54D2) XP®	Cell Signaling Technolo	8243S
Lectin - 488	Vector Laboratories	DL-1174

Supplementary Table 2. Primers used for qPCR

Gene name	Sequence (5' -> 3')
Ptgds_F	GAAGGCGGCCTCAATCTCAC
Ptgds_R	CGTACTCGTCATAGTTGGCCTC
Rps8_F	GCATCTCTCGGGACAACTGG
Rps8_R	CGAGGGCCAATCTTCGTGT
Cst3_F	TGAGCGAGTACAACAAGGGC
Cst3_R	GGCTGGTCATGGAAAGGACAG
Hspa5_F	ACTTGGGGACCACCTATTCCT
Hspa5_R	GTTGCCCTGATCGTTGGCTA
Atp6v0b_F	GCATGGTCGTTGTGGGAATCT
Atp6v0b_R	GGGAAGTTTCCGTCAGGAACC
Rtn1_F	CCGGACCCTCGTTACCAGA
Rtn1_R	TCAGCTAGGATCTTGTTCACCT
Atp6v0c_F	TCGTTTTTCGGTGTCATGGG
Atp6v0c_R	CACTCTTGGCTGTGCCATAGG
Gapdh_F	TGACCTCAACTACATGGTCTACA
Gapdh_R	CTTCCCATTCTCGGCCTTG

Supplementary Table 3: DEGs from snRNA-seq.

This Excel file describes the differentially expressed genes from mouse 5XFAD cortex following sensory stimulation or control. For DEG analysis, the cutoff used in the function FindMarkers in Seurat was: min.pct: 0.25, only test genes that are detected in a minimum fraction of min.pct cells in either of the two populations; logfc.threshold: 0.25.

Supplementary Table 4: Oligonucleotide sequences used to target AQP4

Supplementary Videos

Supplementary Video 1: 2P microscopy z-stacks in 6-month-old 5XFAD mouse prefrontal cortex following no stimulation or 1 hour of noninvasive multisensory gamma stimulation revealing CSF tracer (FITC-Dextran-3kD). CSF tracer (cyan) infused via i.c.m. is labeled in the frontal cortex, and blood vessels (magenta) were labeled with dextran. Volume rendering was performed in Imaris.

Supplementary Video 2: Arterial pulsatility in 6-month-old 5XFAD mouse prefrontal cortex imaged through a cranial window using 2P microscopy and labeled using Texas Red Dextran 70kD. The video depicts change in diameter over time for each vascular segment. Y axis depicts percent change in diameter over baseline diameter; X axis depicts time (minutes); red dot depicts segment of diameter quantification according to the time frame showed in the image.

Supplementary Video 3: Lymphatic vessel volumes in 6-month-old 5XFAD mouse dural meninges imaged using super resolution AiryScan confocal microscopy and visualized in 3D renderings using Imaris. Lymphatic vessels are visualized using Lyve1. Volume renderings were achieved using the Imaris Surfaces feature.

Supplementary Video 4: RNA *in situ* hybridization in 6-month-old 5XFAD mouse prefrontal cortex for *Kcnk1* in *Aldoc*+ cells imaged using confocal microscopy and quantified using 3D renderings and automated spot detection in Imaris. Aldoc is visualized in yellow, Kcnk1 is visualized in cyan, and DAPI is visualized in blue. Volume renderings were achieved using the Imaris Surfaces feature, and spots were achieved using Imaris Spots detection.

Supplementary Video 5: AQP4 immunohistochemistry (magenta) and polarization analysis in 6-month-old 5XFAD mouse prefrontal cortex and visualized in 3D renderings using Imaris. Example images show AQP4 (magenta) and DAPI (blue) visualized in frontal cortex. High resolution imaging reveals AQP4 (magenta) around vascular segment labeled with CD31 (cyan) and eNOS (yellow). Volume renderings were achieved in Imaris Surface detection.

Supplementary Video 6: VIP sensor expressed in mouse culture and visualized before and after administration of VIP receptor agonists. VIP sensor activation signal (green) is quantified over time following bath application of VIP receptor antagonist.
