# Supplementary material for: Multisensory gamma stimulation promotes glymphatic clearance of amyloid
Source: Nature. 2024 Feb 28;627(8002):149–56. doi: 10.1038/s41586-024-07132-6 (PMC10917684; doi:10.1038/s41586-024-07132-6)
Supplement: Supplementary file 2 — Reporting Summary [file 41586_2024_7132_MOESM2_ESM.pdf]

nature portfolio

Corresponding author(s): Li-Huei Tsai

Last updated by author(s): Jan 10, 2024

Reporting Summary

Nature Portfolio wishes to improve the reproducibility of the work that we publish. This form provides structure for consistency and transparency in reporting. For further information on Nature Portfolio policies, see our Editorial Policies and the Editorial Policy Checklist.

Statistics

For all statistical analyses, confirm that the following items are present in the figure legend, table legend, main text, or Methods section.

n/a	Confirmed	
☐	☒	The exact sample size (*n*) for each experimental group/condition, given as a discrete number and unit of measurement
☐	☒	A statement on whether measurements were taken from distinct samples or whether the same sample was measured repeatedly
☐	☒	The statistical test(s) used AND whether they are one- or two-sided<br>*Only common tests should be described solely by name; describe more complex techniques in the Methods section.*
☒	☐	A description of all covariates tested
☐	☒	A description of any assumptions or corrections, such as tests of normality and adjustment for multiple comparisons
☐	☒	A full description of the statistical parameters including central tendency (e.g. means) or other basic estimates (e.g. regression coefficient) AND variation (e.g. standard deviation) or associated estimates of uncertainty (e.g. confidence intervals)
☐	☒	For null hypothesis testing, the test statistic (e.g. *F*, *t*, *r*) with confidence intervals, effect sizes, degrees of freedom and *P* value noted<br>*Give P values as exact values whenever suitable.*
☒	☐	For Bayesian analysis, information on the choice of priors and Markov chain Monte Carlo settings
☒	☐	For hierarchical and complex designs, identification of the appropriate level for tests and full reporting of outcomes
☒	☐	Estimates of effect sizes (e.g. Cohen's *d*, Pearson's *r*), indicating how they were calculated

Our web collection on statistics for biologists contains articles on many of the points above.

Software and code

Policy information about availability of computer code

Data collection	The following softwares were used to collect the data in this study:<br>-Olympus Fluoview (FV31-S, v2.3.1.163) (Olympus Inc)<br>-Zeiss ZEN Blue (v3.3.89) (Carl Zeiss Microscopy)<br>-EthoVision (XT 14) (Noldus)
Data analysis	The following softwares were used to analyze the data in this study<br>-FIJI image processing software (v1.54) (NIH)<br>-Prism (v9.2) (Graph Pad, Inc)<br>-Python (v3.9)<br>-CellRanger (v3.0) (10X Genomics)<br>-Seurat (v4.0.3)<br>-Imaris (v9.1) (Oxford Instruments)<br><br>The code used for the vasomotion analysis can be found at DOI 10.5281/zenodo.10476268. The code used for the snRNA-seq analysis can be found at DOI 10.5281/zenodo.10480275.

For manuscripts utilizing custom algorithms or software that are central to the research but not yet described in published literature, software must be made available to editors and reviewers. We strongly encourage code deposition in a community repository (e.g. GitHub). See the Nature Portfolio guidelines for submitting code & software for further information.

Data

Policy information about availability of data

All manuscripts must include a data availability statement. This statement should provide the following information, where applicable:

- Accession codes, unique identifiers, or web links for publicly available datasets
- A description of any restrictions on data availability
- For clinical datasets or third party data, please ensure that the statement adheres to our policy

All data necessary for the conclusions of this study are presented in the manuscript. snRNA-seq data have been deposited in the Gene Expression Omnibus and can be accessed under accession number GSE249644. The mouse reference genome was used for snRNA-seq (GRCm38 - mm10).

Research involving human participants, their data, or biological material

Policy information about studies with human participants or human data. See also policy information about sex, gender (identity/presentation), and sexual orientation and race, ethnicity and racism.

Reporting on sex and gender	N/A
Reporting on race, ethnicity, or other socially relevant groupings	N/A
Population characteristics	N/A
Recruitment	N/A
Ethics oversight	N/A

Note that full information on the approval of the study protocol must also be provided in the manuscript.

Field-specific reporting

Please select the one below that is the best fit for your research. If you are not sure, read the appropriate sections before making your selection.

☒ Life sciences ☐ Behavioural & social sciences ☐ Ecological, evolutionary & environmental sciences

For a reference copy of the document with all sections, see nature.com/documents/nr-reporting-summary-flat.pdf

Life sciences study design

All studies must disclose on these points even when the disclosure is negative.

Sample size	Sample sizes were chosen on the basis of similar experiments that were previously published (Iaccarino et al., Nature 2016; Martorell et al., Cell 2019; Adaikkan et al., Neuron 2019). Sample sizes were variable in different experiments depending upon the previous experience with the techniques used and availability of age-matched controls. In general, statistical methods were not used to re-calculate or predetermine sample sizes.
Data exclusions	For snRNA-seq, low-quality outlier cells were defined by fewer than 500 or more than 10,000 genes detected, and only genes detected in at least 10 cells were considered. The following quality measures were quantified for each cell: (1) the number of genes for which at least one read was mapped; (2) the total number of counts; (3) the percentage of reads mapped to mitochondrial genes (used to approximate the relative amount of endogenous RNA and commonly used as a measure of cell quality). Cells with a discriminately high ratio of mitochondrial to non-mitochondrial read counts were excluded using unbiased k-means clustering-based binarization. For vasomotion experiments, two-photon microscopy recordings were excluded if motion artifacts prevented reliable image registration. For histology, Grubb's test was used to identify and discard potential outliers. No exclusion criteria were pre-established. One mouse was excluded for EEG analysis due to noisy signal.
Replication	Findings were confirmed with replicate experiments as noted in the figure legends. Measurements and manipulations of glymphatic clearance were obtained using multiple orthogonal approaches.
Randomization	Biological samples were randomly allocated to experimental groups. Animals from different cages but within the same experimental group were selected to assure randomization, such that mice from the same cage received different treatments.
Blinding	Experimenters were blinded to the identity of experimental groups during data analysis but not data acquisition due to the nature of the stimulation. Blinded data analysis of behavioral and immunohistochemistry data was performed using software and scripts.

Reporting for specific materials, systems and methods

We require information from authors about some types of materials, experimental systems and methods used in many studies. Here, indicate whether each material, system or method listed is relevant to your study. If you are not sure if a list item applies to your research, read the appropriate section before selecting a response.

Materials & experimental systems

n/a	Involved in the study
☐	☒ Antibodies
☐	☒ Eukaryotic cell lines
☒	☐ Palaeontology and archaeology
☐	☒ Animals and other organisms
☒	☐ Clinical data
☒	☐ Dual use research of concern
☒	☐ Plants

Methods

n/a	Involved in the study
☒	☐ ChIP-seq
☒	☐ Flow cytometry
☒	☐ MRI-based neuroimaging

Antibodies

Antibodies used

The antibodies are listed in Supplementary Table 1. The following primary antibodies were used in this study: Anti-Aquaporin4 Thermo Fisher Scientific PA5-53234; Anti-CD31 Abcam ab24590; Anti-Connexin 43 Thermo Fisher Scientific 710700; Anti-Cre Recombinase Millipore Sigma MAB3120 clone 7-23; Anti-eNOS Abcam ab76198 (clone 221); Anti-GFAP (2.2B10) Thermo Fisher Scientific 13-0300; Anti-Ly-6A/E Clone E13-161.7 BD Bioscience 553333; Anti-LYVE1 Abcam ab14917; Anti-Mlc1 Thermo Scientific PA5-64327; anti-mouse LYVE-1 Angiobio 11-034; Anti-PECAM-1 Sigma-Aldrich MAB1398Z; Anti-Syntrophin Abcam ab11425; Anti-VIP ImmunoStar 20077; Anti-VIP Antibody (H-6) Santa Cruz Biotechnology sc-25347; Anti-VPAC1 (extracellular) Alomone Labs AVR-001; Anti-β-Amyloid (D54D2) XP® Cell Signaling Technolo 8243S; Lectin - 488 Vector Laboratories DL-1174. All primary antibodies were used at a concentration of 1:500 for immunohistochemistry and 1:50 for TEM. The following secondary antibodies were used: anti-mouse, rat, chicken, goat, rabbit Alexa Fluor 488, 584, or 647 (Thermo Fisher Scientific, all at 1:1000 in PBS-T) raised in donkey or goat. Specifically, goat anti-Rat IgG (H+L) Cross-Adsorbed Secondary Antibody, Alexa Fluor™ 647 ThermoFisher (A21247); Donkey anti-Rabbit IgG (H+L) Highly Cross-Adsorbed Secondary Antibody, Alexa Fluor™ 488 ThermoFisher Scientific A21206; Donkey anti-Rabbit IgG (H+L) Highly Cross-Adsorbed Secondary Antibody, Alexa Fluor™ 488 ThermoFisher A-21206 290; Anti-Rabbit IgG (H+L), highly cross-adsorbed, CF™ 647 antibody produced in goat (SAB4600185).

Validation

Each antibody was validated for the species (mouse) and application (immunohistochemistry) by the corresponding manufacturer.

Eukaryotic cell lines

Policy information about cell lines and Sex and Gender in Research

Cell line source(s)

Human iPSC lines used in this study were generated by the Picower Institute for Learning and Memory iPSC core. For sensor testing, HEK293FT (Invitrogen) and HeLa (ATCC CCL-2) were used.

Authentication

iPSC lines were confirmed by cell marker staining, RNA-sequencing, and karyotyping (see Blanchard et al., 2020). For other cell lines, cells were authenticated by the manufacturer and tested for mycoplasma contamination to their standard levels of stringency.

Mycoplasma contamination

iPSC lines are routinely tested for mycoplasma. For other cell lines, cells were authenticated by the manufacturer and tested for mycoplasma contamination to their standard levels of stringency.

Commonly misidentified lines
(See ICLAC register)

No commonly misidentified lines were used in this study.

Animals and other research organisms

Policy information about studies involving animals; ARRIVE guidelines recommended for reporting animal research, and Sex and Gender in Research

Laboratory animals

Mice were bred in house or obtained from the Jackson Laboratory. Mice were housed in 12-hour light-dark cycle in a temperature- and humidity-controlled environment with water and food ad libitum. All of the animal holding rooms were maintained within temperature (18–26ºC) and humidity ranges (30–70%) described in the ILAR Guide for the Care and Use of Laboratory Animals (1996). Mice were housed in groups no larger than five on a standard 12-hour light/12-hour dark cycle (lights on at 7am; all experiments were performed during the light cycle). The following mouse strains were used in this study: C57l/6J (WT; JAX 000664); 5XFAD (Tg 6799) breeding pairs were acquired from the Mutant Mouse Resource and Research Center (MMRRC) (Jax 034848); Viptm1(cre)Zjh/J (Jax 010908, aka VIP-Cre); VIP-Cre crossed to 5XFAD; VIP-Cre crossed to B6.Cg-Gt(ROSA)26Sortm9(CAG-tdTomato)Hze/J (Jax 007909) to indelibly label VIP interneurons with tdTomato and to generate Ai9/VIP-Cre/5XFAD triple transgenic mice.

Wild animals	This study did not involve wild animals.
Reporting on sex	Male and female mice were used.
Field-collected samples	This study did not involve samples collected from the field.
Ethics oversight	All animal experiments were conducted in accordance with NIH guidelines and were overseen by and adherent to the rules set forth by the Massachusetts Institute of Technology Institutional Animal Care and Use Committee

Note that full information on the approval of the study protocol must also be provided in the manuscript.

Plants

Seed stocks	This study did not involve seed stocks.
Novel plant genotypes	This study did not involve novel plant genotypes.
Authentication	N/A.
